# Triple‐Combination Immunogenic Nanovesicles Reshape the Tumor Microenvironment to Potentiate Chemo‐Immunotherapy in Preclinical Cancer Models

**DOI:** 10.1002/advs.202204890

**Published:** 2023-04-05

**Authors:** Xiaowei Shi, Liwei Shu, Minwen Wang, Jie Yao, Qigu Yao, Suchen Bian, Xiaona Chen, Jianqin Wan, Fu Zhang, Shusen Zheng, Hangxiang Wang

**Affiliations:** ^1^ The First Affiliated Hospital National Health Commission (NHC) Key Laboratory of Combined Multi‐Organ Transplantation Zhejiang University School of Medicine Hangzhou Zhejiang Province 310003 P. R. China; ^2^ Jinan Microecological Biomedicine Shandong Laboratory Jinan Shandong Province 250117 P. R. China; ^3^ Department of Medical Oncology Sir Run Run Shaw Hospital Zhejiang University School of Medicine Hangzhou Zhejiang Province 310016 P. R. China; ^4^ Department of Chemical Engineering Zhejiang University Hangzhou 310027 P. R. China; ^5^ State Key Laboratory for the Diagnosis and Treatment of Infectious Diseases Collaborative Innovation Center for Diagnosis and Treatment of Infectious Diseases The First Affiliated Hospital Zhejiang University School of Medicine Hangzhou 310003 P. R. China; ^6^ National Clinical Research Center for Infectious Diseases Hangzhou 310003 P. R. China

**Keywords:** cancer nanomedicine, chemo‐immunotherapy, gemcitabine prodrug, stimulator of interferon genes agonist, tumor microenvironment

## Abstract

Immune checkpoint blockade (ICB) therapies have had a tremendous impact on cancer therapy. However, most patients harbor a poorly immunogenic tumor microenvironment (TME), presenting overwhelming de novo refractoriness to ICB inhibitors. To address these challenges, combinatorial regimens that employ chemotherapies and immunostimulatory agents are urgently needed. Here, a combination chemoimmunotherapeutic nanosystem consisting of a polymeric monoconjugated gemcitabine (GEM) prodrug nanoparticle decorated with an anti‐programmed cell death‐ligand 1 (PD‐L1) antibody (*α*PD‐L1) on the surface and a stimulator of interferon genes (STING) agonist encapsulated inside is developed. Treatment with GEM nanoparticles upregulates PD‐L1 expression in ICB‐refractory tumors, resulting in augmented intratumor drug delivery in vivo and synergistic antitumor efficacy via activation of intratumor CD8^+^ T cell responses. Integration of a STING agonist into the *α*PD‐L1‐decorated GEM nanoparticles further improves response rates by transforming low‐immunogenic tumors into inflamed tumors. Systemically administered triple‐combination nanovesicles induce robust antitumor immunity, resulting in durable regression of established large tumors and a reduction in the metastatic burden, coincident with immunological memory against tumor rechallenge in multiple murine tumor models. These findings provide a design rationale for synchronizing STING agonists, PD‐L1 antibodies, and chemotherapeutic prodrugs to generate a chemoimmunotherapeutic effect in treating ICB‐nonresponsive tumors.

## Graphical abstract

An immunogenic nanovesicle consisting of a polymeric gemcitabine prodrug nanoparticle decorated with an anti‐programmed cell death‐ligand 1 antibody on the surface and a stimulator of interferon genes agonist encapsulated inside is developed. The vehicle shows targeted delivery of drug payloads and transforms low‐immunogenic tumors into inflamed ones. Systemic administration of this triple‐combination therapy enables sustained antitumor immunity in multiple models.

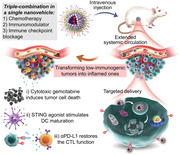

## 1 Introduction

Cancer immunotherapy has shown great potential as a therapeutic paradigm in treating patients with advanced or metastatic cancer recurrence.^[^ ^1^ ^]^ Immune checkpoint blockade (ICB) antibodies that target cytotoxic T‐lymphocyte‐associated protein 4 (CTLA4) or programmed cell death‐1/programmed cell death‐ligand 1 (PD‐1/PD‐L1) constitute an effective regimen and can generate complete and durable responses in cancer patients. Despite these remarkable clinical outcomes, a significant proportion of cancers do not benefit from Food and Drug Administration (FDA)‐approved ICB antibodies.^[^ ^2^ ^]^ Accumulating clinical studies have shown that the lack of efficacy is correlated with a highly immunosuppressed tumor microenvironment (TME), which is characterized by the abundant presence of immunosuppressive cell subtypes together with a paucity of tumor‐infiltrating lymphocytes (primarily CD8^+^ T cells). This immunosuppressive TME may hamper CD8^+^ T‐cell‐mediated antitumor responses and result in refractoriness to ICB therapies. Therefore, to enhance response rates to ICB therapies, innovative solutions that are capable of transforming a low immunogenic TME into a hot TME are essential and urgently needed.^[^ ^3^ ^]^

The stimulator of interferon genes (STING), another independent class of innate immune danger sensors, has emerged as a promising target for cancer immunotherapy. Activation of STING leads to the production of type‐I interferons (IFNs) and other proinflammatory cytokines. These molecules can further promote the maturation of antigen‐presenting cells (APCs), such as dendritic cells (DCs), for priming of de novo CD8^+^ T‐cell responses in vivo, enabling remodeling of the TME as well as the conversion of low immunogenic tumors to hot tumors.^[^ ^4^ ^]^ In this context, previous therapeutic attempts have been made to leverage cyclic dinucleotide (CDN) or nonnucleotide STING agonists to stimulate the innate immune response and to improve the efficacy of ICB therapies. However, CDN‐based STING agents are usually administered via the intratumor injection in current clinical trials, which limits their uses to solid accessible tumors. In addition to these CDN agonists, diamidobenzimidazole (diABZI)‐based compounds (e.g., diABZI‐3) are recently identified synthetic nonnucleotide STING activators. In spite of the potent activity, poor aqueous solubility deters their further clinical implementation.^[^ ^5^ ^]^ Moreover, intravenous administration of small‐molecule STING agonists results in the systemic distribution of these drugs across healthy tissues and insufficient accumulation in the TME. As such, an effective strategy to simultaneously enhance the efficacy and safety of STING agonists is to entrap them in sophisticatedly engineered delivery nanovehicles.

**Figure 1:**
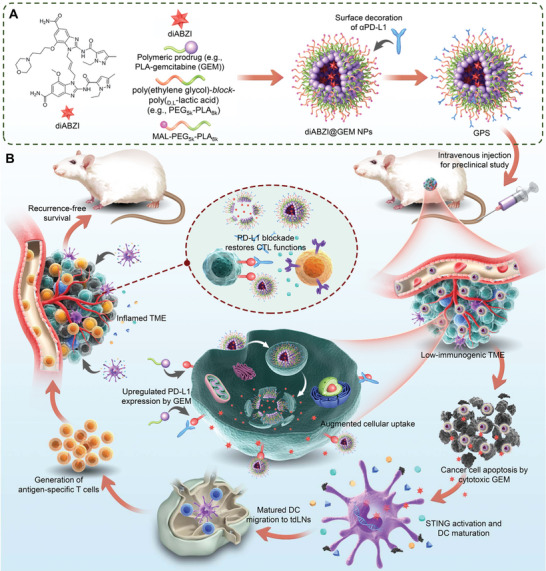
Development of a combinatorial immunogenic nanovesicle for cancer chemoimmunotherapy. A) Schematic illustration showing the preparation of a triple therapy‐integrated nanoparticle composed of a cytotoxic polymeric gemcitabine (GEM) prodrug and a diamidobenzimidazole‐based STING agonist (diABZI) surface‐modified with an anti‐PD‐L1 antibody (*α*PD‐L1). The integrated nanoparticle combining these components is designated GPS based on the concatenated single initials G (GEM), P (*α*PD‐L1), and S (STING agonist). *α*PD‐L1 conjugation not only augments cellular uptake and intratumor drug delivery but also improves the overall antitumor efficacy. B) Upon systemic administration, cytotoxic GEM induces cancer cell apoptosis to generate tumor cell debris, while the diABZI agonist activates the STING signaling pathway to induce DC maturation, cell debris uptake, and cytokine release. These events transform an immunosuppressed “cold” tumor microenvironment (TME) into a “hot” TME and result in robust immune activation, which can further synergize with immune checkpoint blockade with an anti‐PD‐L1 antibody (*α*PD‐L1).

In this study, we generated a triple therapy‐integrated nanoparticle (NP) scaffold that overcomes primary ICB resistance and leads to recurrence‐free survival post‐surgery. We first devised a polylactide (PLA) monoconjugated gemcitabine (GEM) macromolecular prodrug to enhance its metabolic stability and prolong circulation half‐life upon systemic administration.^[^ ^6^ ^]^ GEM is a deoxycytidine analog widely used in the therapy of several forms of advanced pancreatic, lung, breast, ovarian, and bladder cancer.^[^ ^7^ ^]^ However, the therapeutic effects of GEM have been greatly compromised by its short circulation half‐life due to its rapid deactivation and excretion, the development of drug resistance, and its systemic toxicity.^[^ ^6a,b^ ^]^ Encapsulation of a macromolecular GEM conjugate in polymeric micelles benefited in vivo delivery.^[^ ^6c,d^ ^]^ Moreover, exposure to GEM treatment upregulated PD‐L1 expression in tumor cells, which justified the design rationale of using surface‐decorated PD‐L1 antibodies for tumor‐specific drug targeting and delivery, while simultaneously combining with PD‐L1 blockade to improve the overall efficacy. Furthermore, we integrated a STING agonist into GEM‐loaded NPs to generate a triple‐combination immunogenic nanovesicle that reshapes the TME with low immunogenicity. In preclinical mouse models, systemically administered combination nanovesicles not only effectively eradicated large established primary tumors but also enabled long‐lasting rejection of postsurgical tumor recurrence (**Figure** **1**A).

## 2 Results and Discussion

### 2.1 Polymeric GEM Prodrug Synthesis, NP Formulation, and *α*PD‐L1 Decoration

GEM derivatives that are chemically modified with functional moieties at the 4‐(*N*)‐position have been shown to have metabolic stability and prolonged circulation due to the occupation of the deamination site.^[^ ^6c,d^ ^]^ PLA is an FDA‐approved biodegradable polymer that enables intravenous administration and serves as an ideal pro‐moiety for drug derivatization.^[^ ^8^ ^]^ Here, we covalently tethered GEM to the PLA segment to generate the PLA‐GEM conjugate via an amide linkage (Scheme S1, Supporting Information). The final product was readily obtained by purification using silica gel chromatography, with a good yield (≈84%). The structure of the PLA‐GEM prodrug was confirmed by nuclear magnetic resonance (NMR) spectroscopy (^1^H and ^13^C NMR, Figure S1, Supporting Information). The degree of polymerization of PLA in the PLA‐GEM construct was determined to be 22 using end‐group analysis by ^1^H NMR spectroscopy.

**Figure 2:**
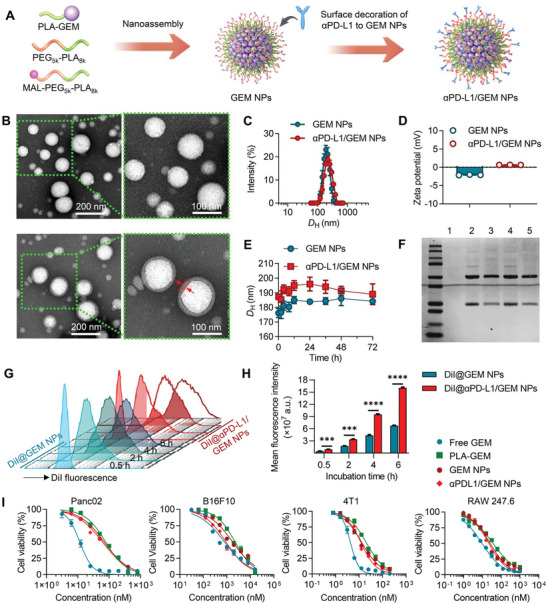
Preparation and characterization of polymeric GEM‐loaded immunogenic NPs. A) Schematic illustration of GEM NP and *α*PD‐L1/GEM NP generation. B) TEM images of GEM NPs (first row) and *α*PD‐L1/GEM NPs (second row). The area enclosed in the green dashed lines was further enlarged and is presented on the right. After antibody conjugation, an electron‐dense shell was observed on the outer layer of *α*PD‐L1/GEM NPs, as marked with red arrows in the enlarged image. The C) Size distribution and D) zeta potential of GEM NPs and *α*PD‐L1/GEM NPs were measured using DLS analysis. E) The *D*_H_ of GEM NPs and *α*PD‐L1/GEM NPs was determined by DLS analysis at different time points. Both NPs were dissolved in saline medium supplemented with 20% FBS to mimic the physiological environment. F) *α*PD‐L1 conjugation was confirmed by Coomassie Brilliant Blue staining. Lane 1: GEM NPs were incubated with iminothiolated *α*PD‐L1 without prior maleimide functionalization; lane 2: resuspended *α*PD‐L1/GEM NPs after ultracentrifugation; lane 3: supernatant solution after ultracentrifugation with excess *α*PD‐L1; lane 4: *α*PD‐L1/GEM NPs before ultracentrifugation; lane 5: iminothiolated *α*PD‐L1 solution used as the positive control. (G‐H) Panc02 cells were treated with DiI@GEM NPs or DiI@*α*PD‐L1/GEM NPs at different time points (e.g., 0.5, 2, 4, and 6 h), and the cellular uptake of both NPs was assessed by flow cytometric analysis. G) The results are presented as histograms, and H) the MFI value of the DiI dye is compared (n = 3). I) Panc02, B16.F10, 4T1, and RAW 247.6 cells were treated with free GEM, the PLA‐GEM conjugate, GEM NPs, and *α*PD‐L1/GEM NPs for 72 h, and cell viability was determined using a CCK‐8 assay. The X‐axis shows the equivalent concentration of GEM (n = 6). The data shown are the mean ± s.e.m. values. Statistical analysis was performed with the Mann–Whitney test. *p<0.05, **p < 0.01, ***p < 0.001 and ****p < 0.0001.

We then formulated the PLA‐GEM conjugate into poly(ethylene glycol)‐*block*‐poly(_D,L_‐lactic acid) (PEG_5k_‐PLA_8k_) polymer NPs for surface PEGylation and solubilization. For further decoration with *α*PD‐L1, terminal maleimide‐functionalized PEG‐PLA (MAL‐PEG_5k_‐PLA_8k_) was employed to conjugate iminothiolated *α*PD‐L1 (**Figure** **2**A). The formation of uniformly spherical nanostructures was verified by TEM observation, with particle sizes of 97 ± 18 and 128 ± 40 nm for GEM NPs and *α*PD‐L1/GEM NPs, respectively (Figure 2B). Upon antibody decoration, an electron‐dense shell was observed on the outer layer of the NPs. The size distributions and zeta potentials of these nanotherapeutics were further evaluated by DLS analysis (Figure 2C,D). The mean hydrodynamic diameter (*D*_H_) of the *α*PD‐L1/GEM NPs was larger than that of the GEM NPs, but the sizes of both NPs ranged between 160–180 nm in DI water. The increased diameter of *α*PD‐L1/GEM NPs could be ascribed to the surface modification of antibodies. To evaluate colloidal stability, saline supplemented with 20% FBS was used to mimic physiological conditions, and a homogeneous size distribution with a small polydispersity index (PDI) for both NPs was observed for up to three days (Figure 2E). Furthermore, the conjugation of *α*PD‐L1 to GEM NPs was confirmed by Coomassie Brilliant Blue staining (Figure 2F). Bands corresponding to the heavy and light chains were observed in the gel lane corresponding to the *α*PD‐L1/GEM NPs. These bands were not observed when GEM NPs without terminal maleimide groups were incubated with iminothiolated *α*PD‐L1. These results further indicated that the antibody was covalently attached but not adsorbed onto the surface.

### 2.2 Increased Cellular Uptake and In Vitro Cytotoxicity of the *α*PD‐L1‐Modified Nanotherapeutic

We assumed that *α*PD‐L1 conjugation to the particle surface could augment the interaction between cancer cells and drug‐loaded NPs, thereby enhancing cellular uptake. To validate this assumption, flow cytometry and confocal laser scanning microscopy (CLSM) were used to assess the cellular uptake of GEM NPs and *α*PD‐L1/GEM NPs. The red fluorescent dye DiI was coencapsulated for NP trafficking. After exposing Panc02 cells to both NPs for different time intervals, flow cytometric analysis was used to quantify the proportion of DiI‐positive cells (Figure 2G). Cellular uptake of the NPs, as indicated by the mean fluorescence intensity (MFI), increased with incubation time (Figure 2H). We further used CLSM to visualize the endocytic process of DiI‐labeled NPs in Panc02 and 4T1 cells. Endo/lysosomal compartments were stained with LysoTracker Green to trace the behavior of NP‐lysosome colocalization following each treatment. The CLSM results showed that both NPs were internalized into cells 2 h after incubation, but cells treated with *α*PD‐L1/GEM NPs exhibited a stronger fluorescence signal (Figure S2, Supporting Information). These data consistently confirmed that NPs conjugated with antibodies are more conducive to phagocytosis.

We next examined the cytotoxicity of these nanotherapeutics in several cancer cell lines, including the pancreatic cancer cell line Panc02, melanoma cell line B16F10, breast cancer cell line 4T1, and macrophage cell line RAW264.7. After exposure to the drugs for 72 h, cell viability was determined using cell counting kit‐8 (CCK‐8) assays. The data showed that the cytotoxic activities of GEM prodrug and related NPs were lower than free GEM across the tested cell lines (Figure 2I). Thus, by conjugating PLA to GEM at the amine position critical for GEM activity, we reduced the in vitro potency of the PLA‐GEM conjugate and related nanoformulations relative to that of the free drug form.

### 2.3 Decoration with *α*PD‐L1 Enhances the Intratumor Drug Delivery of the Nanotherapeutics In Vivo

Encouraged by the enhanced cellular uptake of NPs in cultured cells by *α*PD‐L1 modification, we attempted to explore whether this modification could contribute to increased intratumor drug delivery at tumor sites in animal models. To test this hypothesis, we synthesized a PLA fragment labeled with the near‐infrared fluorescence (NIRF) dye Cy5.5 to form PLA‐Cy5.5 and coassembled the conjugate into *α*PD‐L1/GEM NPs. The NPs were then intravenously injected into C57BL/6 mice bearing Panc02 orthotopic pancreatic tumors (equivalent of 0.75 mg kg^−1^ free Cy5.5 per mouse) for in vivo trafficking. Whole‐body fluorescence imaging was performed under an IVIS Lumina imaging system at different time points after injection. The Cy5.5‐derived NIRF signal rapidly decayed in mice treated with unformulated PLA‐Cy5.5 solution. Encouragingly, a strong signal was maintained in mice injected with Cy5.5‐labeled *α*PD‐L1/GEM NPs during the observation period (Figure S3A, Supporting Information). At 24 h post‐administration, the major organs and tumors were excised and subjected to ex vivo imaging (Figure S3B,C, Supporting Information). Quantitative analysis revealed that in mice treated with Cy5.5‐labeled *α*PD‐L1/GEM NPs, the fluorescence intensities in the major organs were partially reduced, although there were no statistically significant differences in some organs (Figure S3D, Supporting Information). Of note, *α*PD‐L1 modification potentiated the intratumor accumulation; for example, the tumor NIR fluorescence intensities of *α*PD‐L1/GEM NPs‐treated mice were 1.4‐fold higher than those in mice treated with nontargetable GEM NPs and 5.8‐fold higher than those in mice treated with unformulated PLA‐Cy5.5 conjugate (Figure S3E, Supporting Information). This evidence demonstrated that *α*PD‐L1 decoration endowed GEM NPs with preferential accumulation within tumors compared with the accumulation of nontargetable NPs through potential interactions between *α*PD‐L1 and PD‐L1 that is overexpressed on cancer cells.

### 2.4 Antitumor Efficacy and Immunoactivity in an Orthotopic Pancreatic Cancer Model

**Figure 3:**
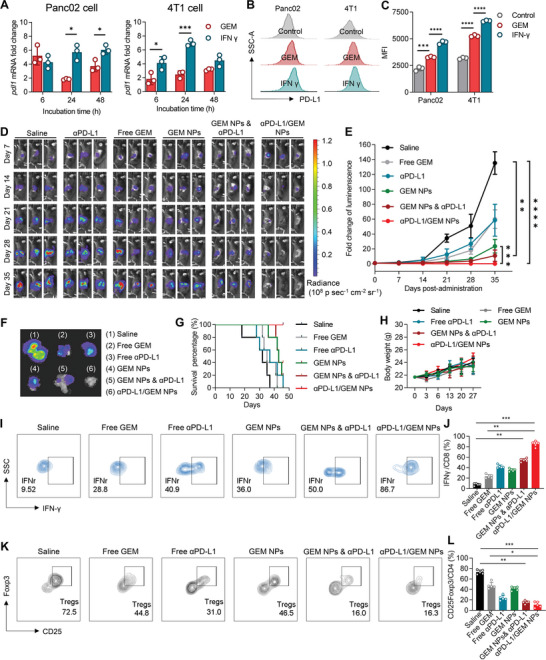
Antitumor efficacy and immunoactivity of *α*PD‐L1/GEM NPs in an orthotopic pancreatic cancer model. A–C) Panc02 and 4T1 cells were treated with 50 nm free GEM or 500 IU mL^−1^ recombinant mouse IFN‐*γ* and incubated for 6, 24, and 48 h. PD‐L1 expression was measured by (A) qPCR and (B and C) flow cytometric analysis. For (B) and (C), the cells were treated with free GEM or IFN‐*γ* for 24 h at the abovementioned concentration. The results are presented as histograms in (B), and the corresponding MFI values are compared in (C). Cells treated with recombinant mouse IFN‐*γ* (500 IU mL^−1^) served as a positive control, as IFN‐*γ* has been reported to upregulate PD‐L1 expression.^[^ ^23^ ^]^ D) In vivo bioluminescence imaging of Panc02‐Luci tumor growth in the different treatment groups (n = 5). E) Tumor growth curves for the different treatment groups (n = 5). Tumor growth was quantified by the total luminescence flux as measured with an IVIS imaging system. The intensity fold change (Y axis) was normalized to the average luminescence flux on day 0. F) On day 35, the mice from different groups were first intraperitoneally injected with _D_‐luciferin at a dose of 75 mg kg^−1^/mouse and then sacrificed. Tumors with pancreatic tissues were harvested for ex vivo inspection. G) Survival curves for the different treatment groups (n = 5). H) Body weight was recorded at different time points. I) Flow cytometry plots and J) statistical chart presenting the proportions of IFN‐*γ* ^+^ CD8a^+^ T cells across different treatment groups. K) Flow cytometry plots and L) statistical chart presenting the proportions of CD25^+^ Foxp3^+^ Treg cells across the different treatment groups. The proportions of the above immune cell subsets were compared across the different treatment groups by the Kruskal–Wallis test. *p<0.05, **p < 0.01, and ***p < 0.001.

For low‐immunogenicity tumors such as pancreatic ductal adenocarcinoma (PDAC), insufficient intrinsic PD‐L1 expression might partially account for the therapeutic failure of ICB monotherapy.^[^ ^9^ ^]^ GEM is the standard chemotherapeutic drug for PDAC treatment in the clinic. Previous studies have shown that treatment with GEM upregulates intratumor PD‐L1 expression in a JAK/Stat1‐dependent manner, resulting in immunosuppression in various tumors.^[^ ^10^ ^]^ Consistent with these studies, we found that PD‐L1 expression was increased in Panc02 and 4T1 cells after exposure to GEM, as determined by quantitative polymerase chain reaction (qPCR) (**Figure** **3**A) and flow cytometric analysis (Figure 3B,C). Given that GEM treatment upregulated PD‐L1 expression in cancer cells, we envisioned that *α*PD‐L1 and GEM could be rationally combined to achieve synergistic therapeutic benefits, which could be additive to the *α*PD‐L1‐mediated tumor targeting and augmented delivery.

Prompted by these results, we first assessed the therapeutic potential of *α*PD‐L1/GEM NPs in a preclinical PDAC model. For this purpose, mouse PDAC Panc02 cells expressing firefly luciferase (Panc02‐Luci) were injected into the pancreatic tail parenchyma of C57BL/6 mice to establish an orthotopic pancreatic cancer model. After two weeks, treatment was initiated. As shown in Figure 3D, intensifying fluorescence signals were observed in the saline‐ and *α*PD‐L1‐treated groups, indicating rapid growth of orthotopic pancreatic tumors. Treatment with GEM, *α*PD‐L1/GEM NPs, and the combination regimen of GEM NPs and *α*PD‐L1 led to relatively attenuated signals until day 28, but tumor relapse occurred on day 35. In sharp contrast, Panc02‐Luci signals were barely detected in *α*PD‐L1/GEM NP‐treated mice, suggesting the eradication of tumor cells. The quantitative fluorescence intensities in each group are presented in Figure 3E and Figure S4, Supporting Information. Not only did treatment with GEM NPs alone show superior tumor inhibition effects over free GEM, but *α*PD‐L1/GEM NPs also had higher efficiency than the combination of GEM NPs and *α*PD‐L1. At the experimental endpoint (day 35), the mice were sacrificed, and the pancreatic tissues were excised for further analyses (Figure 3F). The luciferase intensity was greatly reduced in all treatment groups, but only *α*PD‐L1/GEM NPs completely reduced the tumor burden. Tumor growth was also correlated with survival in mice (Figure 3G). All mice receiving *α*PD‐L1/GEM NPs survived, whereas none of the mice from the other treatment groups remained alive on day 46 post‐inoculation of tumor cells. The stable increase in mouse body weight supported the in vivo safety of the combination therapy (Figure 3H).

Chemotherapy‐induced expression of immunosuppressive factors on cancer cells, such as PD‐L1, can cause the exhaustion and dysfunction of cytotoxic T cells. This phenomenon is classified as acquired immune resistance.^[^ ^10^, ^11^ ^]^ We, therefore, investigated whether additional conjugation of *α*PD‐L1 to GEM NPs would preferentially restore T‐cell immunity in the PDAC TME. For this purpose, tumor infiltrating lymphocytes (TILs) were isolated from pancreatic tumor tissues after three intravenous injections of each agent. Flow cytometry analysis revealed that *α*PD‐L1/GEM NPs markedly increased the proportions of IFN‐*γ* ^+^ CD8^+^ T cells, which represents the activated CD8^+^ T cells against tumor cells, as compared to the combination of GEM NPs with *α*PD‐L1 or *α*PD‐L1 treatment alone (Figure 3I,J). We also quantified the concentration of IFN‐*γ* in tumor homogenates by the ELISA assay. Consistent with the flow cytometry analysis, we found that treatment of *α*PD‐L1/GEM NPs significantly increased the intratumor concentration of IFN‐*γ* (Figure S5A, Supporting Information). Furthermore, the ratio of regulatory T cells (Tregs), an indicator inversely associated with better clinical outcomes,^[^ ^12^ ^]^ was markedly reduced after *α*PD‐L1/GEM NPs treatment (Figure 3K,L and Figure S5B‐E, Supporting Information). These results suggest that particle‐conjugated *α*PD‐L1 preserved its pharmacological effect and GEM chemotherapy was capable of augmenting the *α*PD‐L1 inhibitory activity, while monotherapy with GEM or *α*PD‐L1 failed to reverse the dysfunction of CD8^+^ cytotoxic T lymphocytes (CTLs).

### 2.5 Encapsulation of the STING Agonist into *α*PD‐L1/GEM NPs

**Figure 4:**
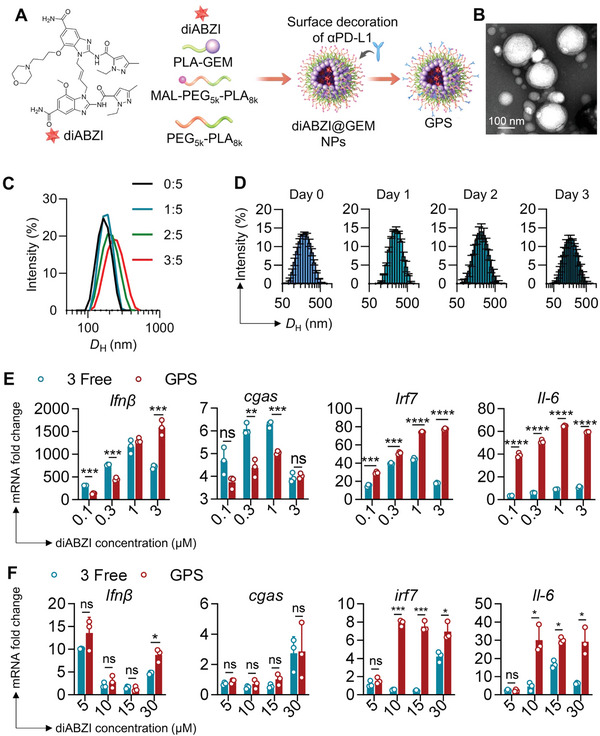
Design and characterization of the GPS therapeutic. A) Schematic illustration of the generation of GPS. B) TEM images of GPS NPs. Scale bars: 100 nm. C) *D*_H_ of GPS as determined by DLS analysis (diABZI was loaded at different mass ratios; the ratios are presented as diABZI:GEM). D) *D*_H_ of GPS NPs (loaded at a mass ratio of 1:5) as determined by DLS analysis. NPs were dissolved in saline medium supplemented with 20% FBS, which mimics the physiological environment. E,F) qPCR analysis of IFN‐*β*, cGAS, IRF7, and IL‐6 gene expression in (E) BMDCs and (F) 4T1 after treatment with diABZIs or a physical mixture of free diABZI, GEM, and *α*PD‐L1 (referred to as 3 free). The X axis shows the equivalent dose of diABZI. The gene expression of Gapdh was defined as an internal reference; all treatment group data were compared to the data in untreated BMDCs or 4T1 (control). Statistical analysis was performed with ANOVA. *p<0.05, **p < 0.01, and ***p < 0.001.

Even though decorated *α*PD‐L1 has been proven to synergize with cytotoxic GEM to elicit robust antitumor efficacy, clinical observations revealed that the majority of cancer patients who lack baseline CD8^+^ T‐cell infiltration at tumor sites are unlikely to respond to ICB therapy.^[^ ^13^ ^]^ In the continuing search for effective immunotherapies, the STING signaling pathway has been suggested to play a critical role in activating the innate immune response. Activation of STING can induce the expression of cytokines, drive DC maturation, and subsequently facilitate T‐cell priming and activation, resulting in immune‐mediated tumor eradication and antitumor immune memory.^[^ ^14^ ^]^ Therefore, we hypothesized that the additional incorporation of a commercially available STING agonist into the *α*PD‐L1/GEM NP platform could improve response rates by transforming low‐immunogenic tumors into inflamed tumors. For this purpose, a diABZI nonnucleotide STING agonist^[^ ^5^ ^]^ was physically encapsulated into the NPs (**Figure** **4**A). To simplify reference to this three‐component nanotherapy, we designated the integrated NP combining these components by concatenating the single initials G (GEM), P (*α*PD‐L1), and S (STING agonist). The resulting GPS nanovesicles showed a spherical‐like structure with a median diameter of ≈180 nm under TEM (Figure 4B), which was consistent with the DLS measurements (Figure 4C). In addition, the STING agonist‐integrated tri‐therapy NPs were colloidally stable in medium containing FBS (Figure 4D). The STING agonist was encapsulated into *α*PD‐L1/GEM NPs with a high efficiency of 63.67%, as determined by high‐performance liquid chromatography (HPLC) analysis. We then investigated whether encapsulation of the diABZI preserved its immunostimulatory potency. DCs are the primary APCs and an important target of adjuvants in maximizing the therapeutic efficacy of vaccines. Mouse bone marrow‐derived DCs (BMDCs), bone marrow‐derived macrophages (BMDMs), and 4T1 breast cancer cells were incubated with GPS or a mixture of free diABZI, GEM, and *α*PD‐L1 (referred to as 3 free), and STING‐associated gene expression was then analyzed by qPCR. Compared to treatment with the free drug combination, GPS therapy led to comparable efficiency of immune activation, with upregulated expression of the STING‐related genes IRF7, IL‐6, and IFN‐*β*. Moreover, the mRNA alteration profiles (Figure 4E,F, Figure S6A, Supporting Information) and ELISA results (Figure S6B, Supporting Information) indicated that nanodelivery of the diABZI in the NPs preserved its high immunostimulatory activity.

### 2.6 In Vivo Blood Circulation, Biodistribution, and Safety Profiles

To examine whether the PLA‐GEM scaffold can prolong the blood circulation of GEM and diABZI after systemic injection, pharmacokinetic analyses were performed on Sprague‐Dawley (SD) rats. Following a single dosing of GPS (15 mg kg^−1^ GEM‐equivalent dose, 3 mg kg^−1^ diABZI, and 200 µg *α*PD‐L1 per rat) or free drug combination, blood samples were collected at different times, and plasma drug concentration over time was determined by HPLC analysis (Figure S7, Supporting Information). Pharmacokinetic parameters extrapolated from the plasma‐drug concentration curves (Figure S7C, Supporting Information) are summarized in Table S1, Supporting Information. The values of area under the concentration‐time curve (AUC_0‐inf_) for GEM and diABZI in GPS were 1381.0±160.4 and 18.6±1.0 µg h mL^−1^, respectively; however, the plasma AUC_0‐inf_ values for free GEM and free diABZI were 411.4±93.2 and 6.2±0.5 µg h mL^−1^, respectively. These data suggest that the GPS platform is capable of extending the systemic circulation of drug payloads in animals, while free drugs are rapidly cleared from the body following intravenous injection. We further analyzed drug tissue biodistribution in the C57BL/6 mouse model of Panc02 orthotopic pancreatic tumors. At 24 h post‐administration, major organs and tumors were excised and drug concentration was measured (Figure S7D, Supporting Information). We found that free drug administration had a statistically significant increase in the accumulation of GEM in kidneys and diABZI in hearts and livers. In contrast, administration of GPS therapy significantly increased drug concentrations in tumors compared to free drugs at an equivalent dose. Preferential accumulation of toxic therapeutics within tumors while sparing healthy organs is a prerequisite to achieving in vivo antitumor activity and reducing side effects.

STING pathway is naturally involved during infection and inflammation. There are concerns that systemic STING activation may exacerbate many autoimmune and inflammatory syndromes.^[^ ^16^ ^]^ We, therefore, investigated the safety profiles of GPS therapy in healthy ICR mice in comparison to its free drug combination. Blood sample analysis and pathological examination of major organs were conducted on days 1 and 30 following the treatment to evaluate acute and long‐term toxicities. In clinical observations, the major side effect of GEM is myelosuppression, which is manifested by leucopenia, thrombocytopenia, and anemia. Consistently, free drug combination resulted in myelosuppression in animals, decreasing the proportions of white blood cells (WBC), neutrophils (NEU), red blood cells (RBC), and platelet (PLT) (Figure S8A, Supporting Information). In contrast, the mice receiving GPS therapy had only mild myelosuppression. Nevertheless, both GPS and 3 free treatment groups showed no evidence of myelosuppression on day 30 (Figure S8B, Supporting Information). In blood biochemistry analyses, administration of free drug combination has only led to a transient elevation of serum alanine aminotransferase (ALT), blood urea nitrogen (BUN), and creatinine (Cre) on day 1 (Figure S8C, Supporting Information), while there were no significant differences in serum aspartate aminotransferase (AST), ALT, BUN and Cre between GPS and saline groups on day 30 (Figure S8D, Supporting Information). In addition, the drug treatment did not impart observable damage to organs, as supported by histopathological examination (Figure S8E,F, Supporting Information). Together, these results suggest that the major toxicity of this combination regime is myelosuppression, and the co‐delivery system can ameliorate bone marrow toxicity compared to the free drug combination.

### 2.7 GPS Therapy Serves as a Neoadjuvant to Inflame the TME and Achieve Recurrence‐Free Survival

**Figure 5:**
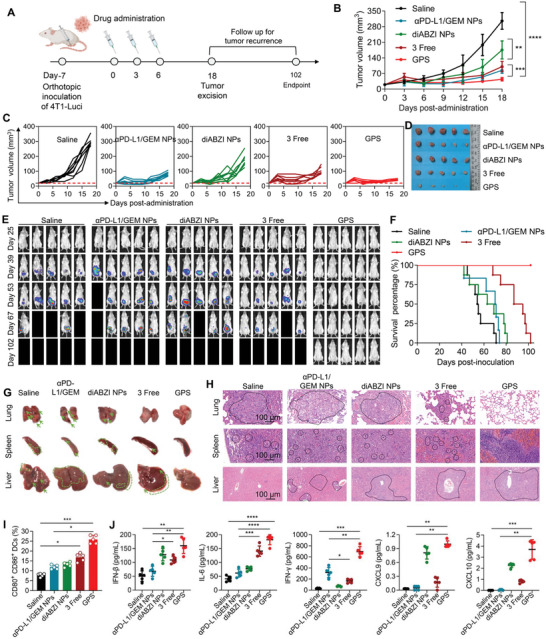
GPS therapy inflamed the TME and achieved recurrence‐free survival in a triple‐negative breast cancer model. A) Schematic of the experimental timeline and the different treatment groups. B,C) Relative tumor volumes were determined at different time points (n = 8). D) Breast cancer tissues were excised on day 18. E) In vivo bioluminescence imaging of 4T1 recurrence and metastasis in different treatment groups (n = 8). F) Survival in different treatment groups (n = 8). G) Gross examination of major organs (lung, liver, and spleen) collected from different treatment groups. Lung metastatic nodules (indicated by the green arrows), liver inflammatory infiltration, splenomegaly, and focal metastasis (indicated by the dashed and arrow‐indicated areas) were observed. H) H&E staining of lungs, spleens, and livers. The dashed areas indicate metastatic lesions in the lungs and spleens and granulocytic infiltration in the livers. Scale bars: 100 µm. I) Flow cytometry plots and statistical chart for CD80^+^ CD86^+^ DCs (distributed in the upper right quadrants in the flow cytometry plots) isolated from TILs. J) IFN‐*β*, IL‐6, IFN‐*γ*, CXCL9, and CXCL10 levels in the 4T1 TME were measured by a bead‐based multiplex LEGENDplex assay. The data shown are the mean ± s.e.m. values (n = 5). Statistical analysis was performed with one‐way ANOVA. *p<0.05, **p<0.01, ***p<0.001, and ****p<0.0001.

Given the critical role of the STING pathway in increasing the responses to immunotherapy, we investigated whether treatment with the STING agonist‐loaded GPS can be applied as neoadjuvant therapy to improve post‐surgical prognosis. The potential of GPS therapy was assessed in a highly aggressive, triple‐negative breast cancer (TNBC) 4T1 model in mice (**Figure** **5**A). After three intravenous injections of each nanotherapeutic, the tumor volume and body weight were monitored. Monotherapy using the diABZI agent (i.e., diABZI NPs) yielded only moderate tumor inhibition. Impressively, GPS therapy successfully induced superior antitumor effects compared to those of the free drug combination (Figure 5B–D). This indicated that the triple therapy‐integrated NPs possessed potent efficacy for reducing the primary tumor burden, which is optimal for complete resection. Moreover, administration of tri‐therapy NPs did not induce apparent body weight loss compared to that in the saline group (Figure S9A,B, Supporting Information) or abnormalities in biochemical indicators in the blood (Figure S9C–F, Supporting Information), suggesting a favorable safety profile in animals.

Metastatic cancer remains a major clinical challenge and is the leading cause of cancer‐related mortality worldwide.^[^ ^17^ ^]^ Considering the highly invasive nature of 4T1 breast cancer, we sought to investigate whether preoperative STING activation would generate a long‐term protective effect against postsurgical tumor recurrence and metastasis. After surgical resection of orthotopic 4T1 breast tumors expressing luciferase (4T1‐Luci) on day 18, in vivo bioluminescence imaging was performed to assess postsurgical tumor recurrence (Figure 5E). Dual therapy (e.g., *α*PD‐L1/GEM NPs) or monotherapy (e.g., diABZI NPs) failed to prevent metastasis, and no mice remained alive on day 102 (Figure 5E,F). Notably, the administration of GPS substantially extended the survival time, with all mice surviving until the endpoint of the study, with no metastatic tumor foci observed by bioluminescence imaging. Gross examination revealed some sentinel signs of tumor metastasis, such as liver inflammatory infiltration and splenomegaly, along with lung metastatic nodules, in the treated mice except for the mice receiving GPS therapy (Figure 5G). Histological analysis using H&E staining also supported these observations, showing a significant distribution of metastatic lesions within the lungs and spleens in the control group mice but no detectable lesions in the GPS‐treated mice (Figure 5H). In addition, mice from the diABZI NPs, *α*PD‐L1/GEM NPs, and saline groups exhibited pronounced granulocyte recruitment to the liver, which was correlated with hepatic metastases.^[^ ^18^ ^]^ These results suggested that activation of the STING pathway synergized with PD‐L1 blockade to potentiate antitumor immunity and that nanodelivery of these therapeutics in a single platform, such as GPS, enabled durable prevention of postsurgical tumor recurrence.

During neoadjuvant downstaging of tumors, cancer cell death results in the exposure of tumor antigens that can be captured and presented by APCs. DCs are the most potent professional APCs, and their functions are tightly regulated by STING activation in a type I IFN‐dependent manner. To gain further insight into the mechanism by which GPS inflames the TME, tumor tissues were harvested on day 8 after the corresponding treatment, and intratumor DCs were isolated for flow cytometric and cytokine/chemokine analyses. We found that GPS therapy significantly stimulated DC maturation, as evidenced by upregulated expression of CD80 and CD86 (Figure 5I and Figure S9G, Supporting Information). We also screened inflammatory cytokine alterations by bead‐based multiplex LEGENDplex immunoassays. Compared with other treatments, administration of GPS resulted in elevated production of IFN‐*β* and IL‐6 in tumor homogenates, which should result from enhanced intratumor delivery of the STING agonist by GPS and, thereby, activation of the STING pathway (Figure 5J). GPS therapy triggered the highest secretion of IFN‐*γ* and tumor necrosis factor *α* (TNF‐*α*), two essential factors for antitumor immunity, in tumors. This therapy also resulted in higher levels of CXCL9 (C‐X‐C motif chemokine ligand, ≈fivefold) and CXCL10 (≈fourfold), which are chemokines that are positively correlated with intratumor T‐cell infiltration, than the free combination. Although diABZI NPs had led to comparable expression levels of IFN‐*β*, CXCL9, and CXCL10 as compared to GPS therapy, the antitumor activity of diABZI NPs as monotherapy was limited. Collectively, these data highlight the potential of the diABZI agent to generate an inflamed TME phenotype that favors efficient killing of primary and micrometastatic lesions for improved recurrence‐free survival. Moreover, triple‐combination therapy using chemotherapy, immune checkpoint inhibition, and STING agonist was potentially effective to provoke strong antitumor activity in low immunogenic tumors.

### 2.8 Tri‐Therapy NPs Eradicate Large Tumors and Elicit Tumor‐Specific Immunity in a Cold Tumor Model

**Figure 6:**
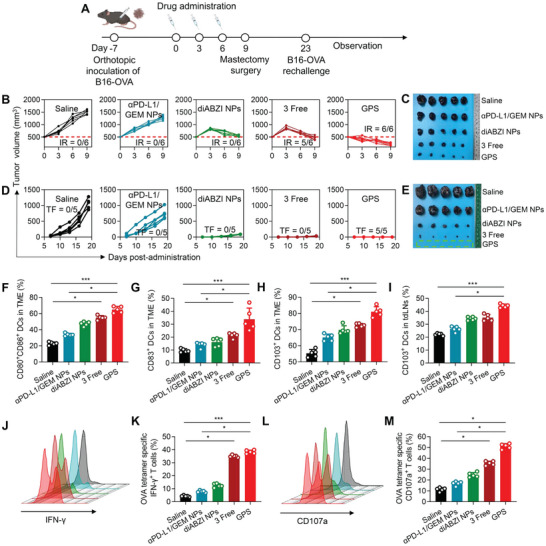
GPS eradicates large tumors and elicits tumor‐specific immunity in an aggressive melanoma model. A) Schematic of the experimental timeline for a rechallenged B16‐OVA melanoma model. B) The relative volumes of primary tumors were determined at different time points (n = 6). “IR” refers to “immune response”, which is defined as the tumor volume on day 9 being smaller than the baseline tumor volume on day 0. C) Primary melanoma tissues were excised on day 9. D) The volumes of rechallenged melanoma tumors were determined at different time points (n = 5). “TF” refers to “tumor free”, which is defined as those without any tumor growth on the contralateral side on day 19 after rechallenge. E) Rechallenged melanoma tissues were excised on day 42. The dashed area indicates that GPS treatment resulted in complete elimination of reinoculated melanoma tumors. F,G) Maturation of DCs, as indicated by the expression levels of CD80, CD86, and CD83, was evaluated in the primary TME, and the results are presented on statistical charts (n = 5). H,I) CD103^+^ DCs were quantified in primary TMEs and tdLNs, and the results are presented in statistical charts (n = 5). J–M) Activation of OVA‐specific CD8+ T cells was evaluated after ex vivo restimulation of splenic lymphocytes with OVA (peptide sequence: SIINFKEL) for 6 h. The proportions of (J, K) IFN‐*γ*+ CD8^+^ T cells and (L, M) CD107a+ CD8^+^ T cells were quantified by flow cytometry. CD107a constitutes a marker of cytotoxic degranulation in CD8^+^ T cells. The proportions of the above immune cell subsets were compared across the different treatment groups by the Kruskal–Wallis test. *p<0.05, **p < 0.01, and ***p < 0.001.

Large tumors remain highly challenging to treat and are often associated with poor survival in the clinic. Even in preclinical animal models, large tumors respond poorly to therapy. We, therefore, sought to determine the efficacy of GPS therapy against aggressive B16‐OVA (B16 cells expressing ovalbumin) melanoma tumors of ≈500 mm^3^ as an inoperable model (**Figure** **6**A). Following systemic administration, the tumor growth curve for each group was recorded (Figure 6B and Figure S10A, Supporting Information). In the saline group, the tumors grew rapidly, and the average tumor volume was 1500 mm^3^ on day 9. However, *α*PD‐L1/GEM NPs without loading of the STING agonist showed low tumor inhibition efficacy, possibly because of the poor immunogenicity of B16F10 melanoma. Strikingly, after three injections of GPS, the large established tumors were significantly eradicated (e.g., the average tumor volume on day 9 was reduced to ≈200 mm^3^). The photograph of tumors excised from each group at the endpoint of the study also supported the efficacy results (Figure 6C). These results indicate that STING agonists are a potential alternative to treat ICB‐resistant tumors and that the use of a neoadjuvant with STING agonists could provide a surgical opportunity for the downstaging of large inoperable malignancies. To further test whether GPS can generate a long‐term durable response, treated mice were rechallenged with the same tumors after surgical removal of the primary melanoma tumors (Figure 6D,E). Rejection of rechallenged tumors was considered evidence of immunological memory. GPS treatment led to responses ranging from robust tumor inhibition to eradication, whereas rapid tumor progression was observed in the saline‐ and dual therapy NP‐treated groups (Figure 6D and Figure S10B, Supporting Information). The tumors excised from each group at the endpoint of the study further demonstrated the superior efficacy of GPS (Figure 6E).

To further elucidate whether GPS therapy induced the systemic immunophenotypic changes, primary tumors, tumor‐draining lymph nodes (tdLNs), and spleens were collected for the isolation of immune cells. Consistent with previous findings, GPS treatment significantly enhanced DC maturation with increased CD80, CD86, and CD83 expression in the TME as compared to other treatments (Figure 6F,G, Figure S10D,E, Supporting Information). The recruitment of tumor‐specific APCs is essential for the initiation of systemic antitumor immune responses, which is followed by DC migration into secondary lymphatic organs. Recent studies have reported the critical role of CD103^+^ DCs in mediating the transport of intact antigens from the TME to tdLNs for the cross‐priming of CD8^+^ T cells, and the expansion of CD103^+^ DCs has offered opportunities for improved cancer immunotherapies with PD‐L1 inhibition in B16 melanoma models.^[^ ^18^ ^]^ Here, we observed that delivery of STING agonists augmented the infiltration of CD103^+^ DCs into both the TME and tdLNs (Figure 6H,I, Figure S10F,G, Supporting Information), indicating successful migration from tumor sites to tdLNs for subsequent cross‐priming. Finally, to determine whether the maturation and migration of DCs established a systemic, antigen‐specific T‐cell response, we analyzed the presence of OVA‐specific CD8^+^ T cells after ex vivo restimulation of splenic lymphocytes with OVA peptide for 6 h (Figure S11, Supporting Information). IFN‐*γ* and CD107a are critical indicators of the activated CD8^+^ effector T cells. As shown by flow cytometric analysis (Figure 6J–M), low levels of OVA‐specific CTLs were detected in the saline and *α*PD‐L1/GEM NP groups but were most abundant in the GPS‐treated group. Notably, monotherapy with the STING agonist failed to elicit a robust OVA‐specific T‐cell response. This was consistent with the current understanding that PD‐1/PD‐L1 signaling among immune cells also affects antitumor responses.^[^ ^19^ ^]^ These data showed that STING agonists can be synergistically combined to sensitize low‐immunogenicity tumors and that GPS therapy can serve as an ideal neoadjuvant for generating systemic, tumor‐specific immunity.

## 3 Conclusion 

In this study, we developed a combinatorial immunogenic nanotherapeutic platform that contains a polymeric monoconjugated GEM prodrug for synergistic delivery of multimodal therapeutics, including chemotherapeutic drugs, ICB inhibitors, and STING agonists. Based on initial pilot findings that GEM treatment upregulates PD‐L1 expression in cancer cells, the chemotherapeutic agent GEM and *α*PD‐L1 were rationally engineered in a single scaffold to enhance cellular uptake and intratumor delivery as well as activate CTLs. To further overcome resistance to ICB and inflame low‐immunogenic tumors, a STING agonist was incorporated to form a triple‐combination GPS nanosystem to synergize innate and adaptive immunity. Neoadjuvant application of GPS induced the maturation of intratumor DCs and upregulated immunostimulatory cytokines and chemokines that are essential for initiating antitumor T‐cell responses. Moreover, GPS therapy efficiently promoted the infiltration and migration of CD103^+^ DCs from the TME to tdLNs, an event considered a bridge between innate and adaptive immunity. In the B16‐OVA rechallenge model, GPS‐pretreated mice showed total rejection of rechallenged tumor cells, and ex vivo experiments revealed that splenic T cells of these mice can generate specific immunity in response to the OVA antigen simulation. As a result, GPS therapy holds promising efficacy in the eradication of both primary tumors and the achievement of recurrence‐free survival in multiple tumor models. Overall, this work provides a framework for devising complementary immunomodulatory agents and chemotherapeutics to synergistically treat ICB‐nonresponsive cancers.

## 4 Experimental Section

### Preparation of Maleimide‐Functionalized GEM NPs

Maleimide‐functionalized PLA‐GEM NPs were fabricated using a nanoprecipitation method. In brief, the PLA‐GEM prodrug, PEG_5k_‐PLA_8k_, MAL‐PEG_5k_‐PLA_8k_, and diABZI were dissolved in 100 µL of DMSO solution and then added dropwise into 900 µL of deionized (DI) water during sonication. NPs were washed three times by repeated ultracentrifugation (35 000 rpm for 1 h at 4 °C) and reconstitution in DI water.

### Iminothiolation of *α*PD‐L1

Iminothiolation of *α*PD‐L1 was conducted according to a previous study^[^ ^20^ ^]^ and the manufacturer's protocol. One milligram of anti‐mouse PD‐L1 antibody (BioXcell Inc., clone 10F.9G2) and 40 µg of 2‐iminothiolane were diluted to a final volume of 0.5 mL in sodium phosphate buffer (pH 8). The mixture was incubated at 4 °C for 2 h in a rotating chamber, followed by repeated centrifugation at 4000 rpm and 4 °C for 30 min using an Ultra‐4 centrifugal filter unit (Amicon, Billerica, MA) to remove excess 2‐iminothiolane.

### Conjugation of the Antibody to Maleimide‐Functionalized GEM NPs

One milligram of iminothiolated *α*PD‐L1was added to 10 mg of maleimide‐functionalized GEM NPs or diABZI@GEM NPs and incubated overnight at room temperature in a rotating shaker. The particles were then pelleted by centrifugation at 14 000 rpm and 4 °C for 30 min and washed twice with DI water. The NP suspension was then lyophilized.

### Cell Lines and Cell Culture

The mouse pancreatic cancer cell line Panc02, mouse melanoma cell line B16.F10 expressing ovalbumin (B16‐OVA), mouse breast cancer cell line 4T1 expressing firefly luciferase (4T1‐Luci), and mouse macrophage cell line RAW 247.6 were obtained from the Cell Bank of the Chinese Academy of Sciences (Shanghai, China) and cultured in DMEM or RPMI‐1640 medium as appropriate. The media were supplemented with 10% fetal bovine serum (FBS), penicillin (100 units mL^−1^), and streptomycin (100 µg mL^−1^).

### Orthotopic Pancreatic Tumor Model

A surgical orthotopic pancreatic cancer model was established in C57BL/6 mice (4–6 weeks old, male).^[^ ^22^ ^]^ Panc02‐Luci cells (5×10^5^) were suspended in 10 µL of PBS and mixed with 10 µL of low‐temperature Matrigel matrix prior to injection into the pancreatic tail parenchyma. Two weeks after inoculation, orthotopically implanted tumors were confirmed with an IVIS Lumina imaging system (Caliper) on day 0, and tumor‐bearing mice were randomly divided into the following six groups (5 mice per group) according to the total luminescence flux: 1) saline (control); 2) *α*PD‐L1 (20 µg per mouse); 3) free GEM (5 mg kg^−1^); 4) GEM NP (5 mg kg^−1^ GEM equivalent); 5) GEM NP (5 mg kg^−1^ GEM equivalent) combined with *α*PD‐L1 (20 µg per mouse), and 6) *α*PD‐L1/GEM NP (equivalent of 5 mg kg^−1^ GEM and 20 µg of *α*PD‐L1 per mouse). The above treatments were intravenously injected every other day for a total of three times. To evaluate anticancer activity and drug toxicity, the tumor burden was monitored by measuring the bioluminescence signals of cancer cells, and the body weight was recorded from day 0 to day 27.

### Orthotopic Breast Cancer Tumor Model

An orthotopic breast cancer model was established in BALB/c mice (4–6 weeks old, female). 4T1‐Luci cells (5×10^5^) were suspended in 50 µL of PBS and inoculated into the mammary fat pad of the mice. Seven days after inoculation, the tumor‐bearing mice were randomly divided into the following five groups and received the designated treatments via intravenous injection every three days for a total of three treatments: 1) saline; 2) diABZI NP; 3) 3 free (physical mixture of free 1 mg kg^−1^ diABZI, 5 mg kg^−1^ GEM and 20 µg of *α*PD‐L1 per mouse); 4) *α*PD‐L1/GEM NP (equivalent of 5 mg kg^−1^ GEM and 20 µg of *α*PD‐L1 per mouse), and 5) GPS (equivalent of 1 mg kg^−1^ diABZI, 5 mg kg^−1^ GEM and 20 µg of *α*PD‐L1 per mouse). The volume of the tumors was calculated using the following formula: Tumor volume = (length*width^2^)/2.

### Orthotopic and Rechallenged Melanoma Model

B16‐OVA cells (5×10^5^ cells) were suspended in 50 µL of PBS and subcutaneously injected into the right axillae of C57BL/6 mice. Seven days after inoculation, when the tumor volume was approximately 500 mm^3^, the mice were randomly divided into the following five groups (5 mice per group) and received treatments via intravenous injection every three days for a total of three treatments: 1) saline; 2) diABZI NP; 3) 3 free (physical mixture of free 1 mg kg^−1^ diABZI, 5 mg kg^−1^ GEM and 20 µg of *α*PD‐L1 per mouse); 4) *α*PD‐L1/GEM NP (equivalent of 5 mg kg^−1^ GEM and 20 µg of *α*PD‐L1 per mouse), and 5) GPS (equivalent of 1 mg kg^−1^ diABZI, 5 mg kg^−1^ GEM and 20 µg of *α*PD‐L1 per mouse). The volume of the tumors was calculated using the following formula: Tumor volume = (length*width^2^)/2. Nine days after the first injection, primary tumors from the same batch of mice were removed and the mice were rechallenged with live B16‐OVA cells on the contralateral side on day 7 after the surgery.

### Cytokine and Chemokine Analysis

Tumors were harvested 2 days after the last injection of the corresponding treatment. Tumor tissues (approximately 2 mg) were excised and minced in 300 µL of RIPA lysis buffer (Beyotime, catalog no. P0013B) supplemented with protease and phosphatase inhibitor cocktail (Beyotime, catalog no. P1046). The protein content was first quantified with a BCA protein assay kit (Beyotime, catalog no. P0012S).

The levels of cytokines and chemokines in the tumor tissue homogenates were measured with a LEGENDplex Mouse Inflammation Panel (13‐plex) (catalog no. 740446), LEGENDplex Mouse Proinflammation Chemokine Panel (13‐plex) (catalog no. 740451), and Mouse IFN‐*β* Valukine ELISA Kit (Novus, Bio‐Techne China, catalog no. VAL612). The resulting values were then normalized to the protein content as determined by the BCA.

### TAA‐Specific T‐Cell Analysis

Spleens from B16‐OVA tumor‐bearing C57BL6/J mice were collected 2 days after the last injection of the corresponding treatment, homogenized into single‐cell suspensions, and then treated with ACK Lysing Buffer (Gibco). A total of 2 × 10^6^ splenocytes were seeded in a 6‐well plate in 1 mL of DMEM containing 10% FBS and supplemented with H‐2K^b^ OVA peptide (5 g mL^−1^, catalog no. 257–264) (MBL Life Science, catalog no. TS‐5001‐P) for 6 h. Finally, the cells were stained with dye‐labeled antibodies and subjected to flow cytometry analysis following the manufacturer's protocol.

### Animal Experiments

C57BL/6 mice, BALB/c mice, ICR mice and Sprague‐Dawley (SD) rats were purchased from the Laboratory Animal Center of Hangzhou Medical College (Hangzhou, China). All animals were raised in specific pathogen‐free animal experimental center and were allowed free access to food and water. All animal studies were conducted in accordance with the National Institute Guide for the Care and Use of Laboratory Animals. The protocols for animal experiments were approved by the Ethics Committee of the First Affiliated Hospital, Zhejiang University School of Medicine.

### Statistical Analyses

Quantitative data were presented as the means ± standard deviations. Data were analyzed using SPSS 22.0 software. Significant differences were analyzed using one‐way ANOVA and the chi‐square test. The thresholds for statistical significance were *p<0.05, **p<0.01, and ***p<0.001.

## Conflict of Interest

The authors declare no conflict of interest.

## Supporting information

Supporting Information

## Data Availability

The data that support the findings of this study are available from the corresponding author upon reasonable request.

## References

[1] a) A. D. Waldman , J. M. Fritz , M. J. Lenardo , Nat. Rev. Immunol. 2020, 20, 651; 32433532 10.1038/s41577-020-0306-5 PMC7238960

[2] a) A. Ribas , J. D. Wolchok , Science 2018, 359, 1350; 29567705 10.1126/science.aar4060 PMC7391259

[3] a) J. Wan , L. Ren , X. Li , S. He , Y. Fu , P. Xu , F. Meng , S. Xian , K. Pu , H. Wang , Proc. Natl. Acad. Sci. U. S. A. 2023, 120, e2210385120; 36787350 10.1073/pnas.2210385120 PMC9974508

[4] a) M. B. Fuertes , A. K. Kacha , J. Kline , S.‐R. Woo , D. M. Kranz , K. M. Murphy , T. F. Gajewski , J. Exp. Med. 2011, 208, 2005; 21930765 10.1084/jem.20101159 PMC3182064

[5] J. M. Ramanjulu , G. S. Pesiridis , J. Yang , N. Concha , R. Singhaus , S. Y. Zhang , J. L. Tran , P. Moore , S. Lehmann , H. C. Eberl , M. Muelbaier , J. L. Schneck , J. Clemens , M. Adam , J. Mehlmann , J. Romano , A. Morales , J. Kang , L. Leister , T. L. Graybill , A. K. Charnley , G. Ye , N. Nevins , K. Behnia , A. I. Wolf , V. Kasparcova , K. Nurse , L. Wang , A. C. Puhl , Y. Li , et al., Nature 2018, 564, 439. 30405246 10.1038/s41586-018-0705-y

[6] a) Y. Binenbaum , S. Na'ara , Z. Gil , Drug Resistance Updates 2015, 23, 55; 26690340 10.1016/j.drup.2015.10.002

[7] a) H. A. 3rd Burris , M. J. Moore , J. Andersen , M. R. Green , M. L. Rothenberg , M. R. Modiano , M. C. Cripps , R. K. Portenoy , A. M. Storniolo , P. Tarassoff , R. Nelson , F. A. Dorr , C. D. Stephens , D. D. Von Hoff , J. Clin. Oncol. 1997, 15, 2403; 9196156 10.1200/JCO.1997.15.6.2403

[8] B. Tyler , D. Gullotti , A. Mangraviti , T. Utsuki , H. Brem , Adv. Drug Delivery Rev. 2016, 107, 163. 10.1016/j.addr.2016.06.018 27426411

[9] L. Zheng , J. Natl. Cancer Inst. 2017, 109, djw304. 28131993 10.1093/jnci/djw304 PMC5291186

[10] a) H. Wang , X. He , D. Fang , X. Wang , J. Guan , Z. W. Shi , X. Chen , Clin. Res. Hepatol. Gastroenterol. 2021, 46, 101853; 34923183 10.1016/j.clinre.2021.101853

[11] K. C. Huang , S. F. Chiang , W. T. Chen , T. W. Chen , C. H. Hu , P. C. Yang , T. W. Ke , K. S. C. Chao , Cancers 2020, 12, 462. 32079180 10.3390/cancers12020462 PMC7072566

[12] Y. Togashi , K. Shitara , H. Nishikawa , Nat. Rev. Clin. Oncol. 2019, 16, 356. 30705439 10.1038/s41571-019-0175-7

[13] a) A. C. Diederichsen , J. Hjelmborg , P. B. Christensen , J. Zeuthen , C. Fenger , Cancer Immunol. Immunother. 2003, 52, 423; 12695859 10.1007/s00262-003-0388-5 PMC11032970

[14] R. A. Watson , O. Tong , R. Cooper , C. A. Taylor , P. K. Sharma , A. V. d. l. Aires , E. A. Mahé , H. Ruffieux , I. Nassiri , M. R. Middleton , B. P. Fairfax , Sci. Immunol. 2021, 6, eabj8825. 34597125 10.1126/sciimmunol.abj8825 PMC7612602

[15] a) L. Corrales , L. H. Glickman , S. M. McWhirter , D. B. Kanne , K. E. Sivick , G. E. Katibah , S. R. Woo , E. Lemmens , T. Banda , J. J. Leong , K. Metchette , T. W. jr Dubensky , T. F. Gajewski , Cell Rep. 2015, 11, 1018; 25959818 10.1016/j.celrep.2015.04.031 PMC4440852

[16] A. Decout , J. D. Katz , S. Venkatraman , A. Ablasser , Nat. Rev. Immunol. 2021, 21, 548. 33833439 10.1038/s41577-021-00524-z PMC8029610

[17] a) Y. Sun , X. Feng , C. Wan , J. F. Lovell , H. Jin , J. Ding , Asian J. Pharm. Sci. 2021, 16, 129; 33995609 10.1016/j.ajps.2020.05.004 PMC8105413

[18] S. Tabariès , V. Ouellet , B. E. Hsu , M. G. Annis , A. A. N. Rose , L. Meunier , E. Carmona , C. E. Tam , A.‐M. Mes‐Masson , P. M. Siegel , Breast Cancer Res. 2015, 17, 45. 25882816 10.1186/s13058-015-0558-3 PMC4413545

[19] a) E. W. Roberts , M. L. Broz , M. Binnewies , M. B. Headley , A. E. Nelson , D. M. Wolf , T. Kaisho , D. Bogunovic , N. Bhardwaj , M. F. Krummel , Cancer Cell 2016, 30, 324; 27424807 10.1016/j.ccell.2016.06.003 PMC5374862

[20] Q. Peng , X. Qiu , Z. Zhang , S. Zhang , Y. Zhang , Y. Liang , J. Guo , H. Peng , M. Chen , Y. X. Fu , H. Tang , Nat. Commun. 2020, 11, 4835. 32973173 10.1038/s41467-020-18570-x PMC7518441

[21] S. K. Swaminathan , E. Roger , U. Toti , L. Niu , J. R. Ohlfest , J. Panyam , J Control Release 2013, 171, 280. 23871962 10.1016/j.jconrel.2013.07.014

[22] a) L. I. Partecke , M. Sendler , A. Kaeding , F. U. Weiss , J. Mayerle , A. Dummer , T. D. Nguyen , N. Albers , S. Speerforck , M. M. Lerch , C. D. Heidecke , W. von Bernstorff , A. Stier , Eur. Surg. Res. 2011, 47, 98; 21720167 10.1159/000329413

[23] a) K. Abiko , N. Matsumura , J. Hamanishi , N. Horikawa , R. Murakami , K. Yamaguchi , Y. Yoshioka , T. Baba , I. Konishi , M. Mandai , Br. J. Cancer 2015, 112, 1501; 25867264 10.1038/bjc.2015.101 PMC4453666

