# Supplementary material for: Triple‐Combination Immunogenic Nanovesicles Reshape the Tumor Microenvironment to Potentiate Chemo‐Immunotherapy in Preclinical Cancer Models
Source: Adv Sci (Weinh). 2023 Apr 5;10(15):2204890. doi: 10.1002/advs.202204890 (PMC10214259; doi:10.1002/advs.202204890)
Supplement: Supplementary file 1 — Supporting Information [file ADVS-10-2204890-s001.pdf]

Supporting information

Triple-Combination Immunogenic Nanovesicles Reshape the Tumor Microenvironment to Potentiate Chemo-Immunotherapy in Preclinical Cancer Models

Xiaowei Shi[1,2], Liwei Shu[3], Minwen Wang [3], Jie Yao[1,4], Qigu Yao[5,6], Suchen Bian[1], Xiaona Chen[1,2], Jianqin Wan[1,2], Fu Zhang[1], Shusen Zheng*[1,2] and Hangxiang Wang*[1,2]

[1] The First Affiliated Hospital, NHC Key Laboratory of Combined Multi-Organ Transplantation, Zhejiang University School of Medicine, Hangzhou, Zhejiang Province, 310003, PR China

[2] Jinan Microecological Biomedicine Shandong Laboratory, Jinan, Shandong Province, 250117, PR China

[3] Department of Medical Oncology, Sir Run Run Shaw Hospital, Zhejiang University School of Medicine, Hangzhou, Zhejiang Province, 310016, PR China

[4] Department of Chemical Engineering, Zhejiang University, Hangzhou 310027, PR China

[5] State Key Laboratory for the Diagnosis and Treatment of Infectious Diseases, Collaborative Innovation Center for Diagnosis and Treatment of Infectious Diseases, The First Affiliated Hospital, Zhejiang University School of Medicine, Hangzhou 310003, PR China

[6] National Clinical Research Center for Infectious Diseases, Hangzhou 310003, PR China

***Corresponding Authors:**

i) Shusen Zheng, M.D., Ph.D. Division of Hepatobiliary and Pancreatic Surgery, Department of Surgery, The First Affiliated Hospital, Zhejiang University School of Medicine, 79 Qingchun Rd., Hangzhou 310003, China.

E-mail: shusenzheng@zju.edu.cn

ii) Hangxiang Wang, Ph.D. Division of Hepatobiliary and Pancreatic Surgery, The First Affiliated Hospital, Zhejiang University School of Medicine, 79 Qingchun Road, Hangzhou 310003, China.

E-mail: wanghx@zju.edu.cn

Materials and Methods

Gemcitabine (GEM), triethylene glycol monomethyl and 2-iminothiolane hydrochloride were purchased from Aladdin Holding's Group Co., Ltd (Shanghai, China). D,L-Lactide was purchased from Servicebio Biotechnology Co., Ltd (Wuhan, China). Anti-mouse PD-L1 antibody (clone:10F.9G2) was purchased from BioXCell company (USA). $mPEG_{5k}$-PLA_{8k} was obtained from Daigang Biomaterial Co., Ltd (Jinan, China). Maleimide-functionalized PEG_{5k}-PLA_{8k} (MAL-PEG_{5k}-PLA_{8k}) was purchased from Ruixi Biotechnology Co., Ltd (Xi'an, China). STING agonist-3 (diABZI) was purchased from MedChemExpress (USA). LEGENDplex mouse inflammation panel (13-plex) was purchased from BioLegend (USA). DiR dye was purchased from Thermofisher (USA). Lysotracker Green and D-Luciferin were purchased from Yeason Biotechnology Co., Ltd (Shanghai, China). The cell counting kit-8 (CCK-8) was purchased from Dojindo China Co., Ltd. (Shanghai, China). All other compounds and solvents were purchased from commercial suppliers (Sigma-Aldrich, Tokyo Chemical Industry (TCI) or J&K Chemical) and used without further purification.

All reactions were performed in a dry atmosphere. Thin layer chromatography (TLC) was performed on silica gel 60 F_{254} precoated aluminium sheets (Merck). Flash column chromatography on neutral silica gel (Qingdao Haiyang Chemical Co., Ltd) was used for compound purification. ^{1}H and ^{13}C nuclear magnetic resonance (NMR) spectra were recorded on a Bruker 400 spectrometer and analyzed with MestReNova software (v12.0.0-20080, Mestrelab Research S.L.). Chemical shifts were calibrated to the residual solvent peak or tetramethylsilane (= 0 ppm). Multiplicities are abbreviated as follows: s = singlet, d = doublet, t =triplet, q = quartet, m = multiplet, dd = double doublet. Ultraviolet-visible (UV-vis) absorption spectra were taken on UV-2700 spectrometer (Shimadzu). Transmission electron microscopy (TEM) images were captured by Tecnai G2 spirit 120 kV transmission microscope (Thermo FEI). Confocal images were captured on IX83-FV3000-OSR laser confocal microscopy (Olympus). In vivo fluorescence images were acquired through IVIS Lumina LT spectrum imaging system (PerkinElmer). Tissue sections were digitized by NanoZoomer digital pathology system (Hamamatsu).

Synthesis of Gem-Boc

Gemcitabine (GEM) (263.2 mg, 1.0 mmol) and di-tert-butyl dicarbonate (436.5 mg, 2.0 mmol) were mixed and dissolved in 40 mL anhydrous *N*,*N*-dimethylformamide (DMF). The mixture was subsequently stirred at 45°C under nitrogen atmosphere overnight. Finally, light yellow syrupy liquid Gem-Boc (78.1 mg) was obtained after column chromatography purification, with a yield of 16.9%. ^{1}H NMR (400 MHz, Chloroform-*d*) δ 7.47 (d, J = 7.5 Hz, 1H), 6.42 (d, J = 10.0 Hz, 1H), 5.82 (d, J = 7.5 Hz, 1H), 5.12 (s, 1H), 4.55 – 4.20 (m, 3H), 1.83 (s, 2H), 1.83 – 1.13 (m, 18H).

^{13}C NMR (100 MHz, DMSO-d_6) δ 166.12, 155.15, 146.70, 141.25, 123.58, 95.05, 86.10, 80.83,

69.11, 59.40, 27.32.

Synthesis of PLA-GEM-Boc

Carboxyl-terminated polylactide (PLA) (100.0 mg, 54.1 μmol) and Gem-Boc (25.1 mg, 54.1 μmol) were dissolved in 20 mL anhydrous dichloromethane (DCM). *N*-Ethyl-*N*'-(3-dimethylaminopropyl)carbodimide (EDC, 20.0 μL, 113.0 mmol) was then added dropwise under agitation. The mixture was subsequently stirred at 37°C under a nitrogen atmosphere overnight. Finally, PLA-GEM--Boc (117 mg) was obtained after column chromatography purification, with a yield of 94.2%. ^{1}H NMR (400 MHz, Chloroform-*d*) δ 7.81 (d, *J* = 7.7 Hz, 1H), 7.43 (d, *J* = 7.7 Hz, 1H), 6.69 – 6.29 (m, 1H), 5.48 – 4.97 (m, 23H), 4.72 – 4.16 (m, 5H), 3.74 – 3.51 (m, 10H), 3.38 (s, 3H), 2.79 (d, *J* = 5.2 Hz, 4H), 1.71 (s, 1H), 1.65 – 1.53 (m, 66H), 1.51 (d, *J* = 3.4 Hz, 18H).

^{13}C NMR (100 MHz, DMSO-d_6) δ 174.55, 174.12, 173.62, 170.50 – 169.35, 169.02, 166.11, 155.13, 146.70, 141.26, 126.54 – 120.86, 95.03, 86.10, 80.82, 72.63, 71.73, 70.35 – 69.91, 69.69 – 68.23, 65.99, 64.75, 59.40, 58.52, 29.24, 27.32, 20.84, 16.97, 15.57.

Synthesis of the PLA-GEM conjugate

PLA-GEM-Boc (50.0 mg, 21.8 μmol) was dissolved in the mixture solution of 10 mL DCM and 10 mL trifluoroacetic acid (TFA). The mixture was subsequently stirred at 37°C under nitrogen atmosphere for 3 h. Finally, PLA-GEM (38.6 mg) was obtained after vacuum drying without further purification, with a yield of 84.5%. ^{1}H NMR (400 MHz, Chloroform-*d*) δ 8.38 (s, 1H), 7.45 (s, 1H), 6.18 (s, 1H), 5.44 – 5.01 (m, 22H), 4.60 – 4.43 (m, 1H), 4.38 – 4.19 (m, 2H), 3.99 (d, *J* = 44.0 Hz, 3H), 3.77 – 3.49 (m, 10H), 3.38 (s, 3H), 2.84 (d, *J* = 33.8 Hz, 4H), 2.24 (t, *J* = 7.6 Hz, 1H), 2.00 (t, *J* = 6.4 Hz, 1H), 1.72 (d, *J* = 7.3 Hz, 1H), 1.57 (m, 66H).

^{13}C NMR (100 MHz, DMSO-d_6) δ 174.55, 174.12, 173.62, 170.54 – 169.28, 169.01, 166.11, 155.13, 141.28, 126.51 – 120.18, 95.03, 80.84, 72.63, 71.73, 70.20, 70.07, 69.69 – 68.97, 68.61, 65.93, 64.75, 59.40, 58.52, 30.68 – 27.73, 20.83, 17.06, 15.57.

Characterization of nanoparticle size and zeta potential

The hydrodynamic diameter (D_H), polydispersity index (PDI), and ζ potential of the drug-loaded micelles were analyzed by DLS using a Malvern Nano-ZS 90 laser particle size analyzer (Malvern Instruments, Malvern, UK) at 25°C.

Transmission electron microscopy analysis

The morphological characteristics of the nanoparticles were observed by TEM (Philips, 120 kV, Netherlands). GEM NPs, αPD-L1/GEM NPs and GPS were prepared at 1 mg/mL GEM-equivalent concentration. A droplet of each solution was dripped on a 400 mesh copper grid. After 5 min of deposition, the excess water on the surface was absorbed by a filter paper and dried in the air. Aqueous solution containing 2 wt % uranyl acetate was used for positive staining.

Quantitative real time PCR (qRT-PCR) assay

Total RNA was extracted using Trizol reagent (Takara, Dalian, China). Then cDNA was obtained by reverse transcription using a cDNA transcription kit (Takara, Dalian, China) according to manufacturer's instructions. Quantitative real time PCR (qRT-PCR) was performed using SYBR Green mix from Takara on Light Cycler 96 (Roche, USA). β-actin was selected as internal control. Primer sequences were listed as follows:

PDL1 forward 5'-TGCGGACTACAAGCGAATCACG-3'

reverse 5'-CTCAGCTTCTGGATAACCCTCG-3'

IFN--β forward 5'-CAGCTCCAAGAAAGGACGAAC-3'

reverse 5'-GGCAGTGTAACTCTTCTGCAT-3'

cGAS forward 5'-TTCCACGAGGAAATCCGCTGAG-3'

reverse 5'-CAGCAGGGCTTCCTGGTTTTTC-3'

IRF7 forward 5'-ATGCACAGATCTTCAAGGCCTGGGC-3'

reverse 5'-GTGCTGTGGAGTGCACAGCGGAAGT-3'

IL-6 forward 5'-TACCACTTCACAAGTCGGAGGC-3'

reverse 5'-CTGCAAGTGCATCATCGTTGTTC-3';

For qPCR analysis of BMDCs or BMDMs, the cells were first seed at a concentration of 10^4 cells/ well in 6-well plates. After 24 hours of adherence, the cells were treated with GPS or 3 Free for 4 hours. Gene expression of Gadph was defined as internal reference; all treatments were compared to untreated BMDCs or BMDMs (control).

For qPCR analysis of 4T1 cells, the tumor cells were first seed at a concentration of 10^4 cells/ well in 6-well plates. After 24 hours of adherence, the cells were treated with GPS or 3 Free for 3 hours. Gene expression of Gadph was defined as internal reference; all treatments were compared to untreated 4T1 (control).

In vivo bioluminescence analysis

Mice bearing Panc02-Luci pancreatic cancer or 4T1-Luci breast tumor cancer were injected intraperitoneally with 0.1 ml of D-luciferin at a dose of 75mg/kg per mouse) and were then anesthetized with 1.5% isoflurane in oxygen in a ventilated anesthesia chamber and imaged 7 min after the injection with an in vivo imaging system (IVIS, PerkinElmer).

Pharmacokinetic assay

Sprague-Dawley (SD) rats were randomly divided into two groups (4 rats per group) and treated as follows: (1) GPS (15 mg/kg GEM-equivalent dose, 3 mg/kg diABZI, and 200 µg αPD-L1 per rat) or (2) free drug combination. Prior to administration, blood was collected from each rat as baseline sample. Following drug administration, blood samples were collected at predetermined time intervals (5 min, 30 min, 1 h, 2 h, 4 h, 6 h, 24 h and 48 h). All blood samples were collected in anticoagulant tubes and centrifuged for 10 min at 12,000 rpm to obtain serum. All samples were treated with sodium hydroxide (0.1 mol L^{-1}) for 2 h and neutralized with

hydrogen chloride. Finally, the hydrolyzed sample was extracted by acetonitrile, and the drug concentration was determined by analytical high-performance liquid chromatography (HPLC). Drug concentrations were quantified using a standard curve. HPLC was carried out on a Hitachi Chromaster system, and a YMC-Pack ODS-A C18 column (5 μm, 250 × 4.6 mm) at a flow rate of 1.0 mL/min. The UV detection wavelengths for GEM and diABZI are 280 nm and 325 nm, respectively.

Biodistribution study

Panc02 orthotopic pancreatic model was used for investigation of drug tissue biodistribution. The mice were randomly divided into two groups (4 mice per group) and treated with a single injection as follows: (1) GPS (15 mg/kg GEM-equivalent dose, 3 mg/kg diABZIs and 20 μg αPD-L1 per mouse); (2) free drug combination at the same doses of each drug. At 24 h post-injection, the mice were sacrificed, and major organs and primary tumors were collected. The tissues were frozen in liquid nitrogen and manually pulverized with a mortar and pestle. All samples were treated with sodium hydroxide (0.1 mol L^{-1}) for 2 h and neutralized with hydrogen chloride. Finally, the hydrolyzed tissue samples extracted by acetonitrile and subjected to HPLC analysis.

Isolation of tumor-infiltrating leukocytes

After 48h of the third injection, the mice were sacrificed and tumors were collected. The tumors were snipped into multiple pieces and digested in thermo shaker at 37°C for 30 min. The digested tissues were then squashed by the plunge of syringes through cell strainers (70 μm). The cell suspension was centrifuged at 400 g for 5 min. The supernatant was discarded and the cells were resuspended in 4 ml of RPMI. 3 ml of 100% Ficoll-hypaque, 3ml of 75% Ficoll-Hypaque and 4ml of cell suspension was layered in sequence for density gradient centrifugation (300 g for 30 min). Tumor-infiltrating leukocytes were harvested between the two layers of Ficoll-hypaque for flow cytometry analysis.

To analyze T cell populations, TILs were stained with BV605 anti-mouse CD45 (BioLegend, clone 30-F11, catalog no. 103155); FITC anti-mouse CD3e (BioLegend, clone17A2, catalog no. 100203); PE/Cy7 anti-mouse CD4 (BioLegend, clone GK1.5, catalog no. 100422); Pacific Blue anti-mouse CD8a (BioLegend, clone 53-6.7, catalog no. 100725); APC anti-mouse IFN-γ (BioLegend, clone XMG1.2, catalog no. 505810); BV785 anti-mouse CD25 (BioLegend, clone PC61, catalog no. 102051); PE anti-mouse Foxp3 (BioLegend, clone 150D, catalog no. 320008) and Zombie Aqua™ (BioLegend, catalog no. 423102). The gating strategy and CD8/CD4 plots for each group was provided in Figure S4 B to E.

To analyze the DC maturation, TILs were stained with Alexa Fluor 700 anti-mouse CD45 (eBioscience, catalog no. 56-9459-42); PE/Cy7 anti-mouse CD11c (BioLegend, clone N418, catalog no. 117318); Brilliant Violet 605 anti-mouse F4/80 (BioLegend, clone BM8, catalog

no. 123133); Brilliant Violet 421 anti-mouse I-A/I-E (BioLegend, clone M5/114.15.2, catalog no. 107632); Brilliant Violet 711 anti-mouse CD103 (BioLegend, clone 2E7 catalog no. 121435); PerCP/Cy5.5 anti-mouse CD86 (BioLegend, clone GL-1, catalog no. 105028), Brilliant Violet 650 anti-mouse CD80 (BioLegend, clone 16-10A1, catalog no. 104731), Zombie NIR Fixable Viability Kit (Biolegend, catalog 423105). The gating strategy was provided in Figure S10C.

Quantification of IFN-γ in pancreatic tumor tissues

An orthotopic pancreatic tumor model was established according to the protocol described in the experimental section (orthotopic pancreatic tumor model). After 2 weeks of inoculation, the tumor-bearing mice were randomly divided into the following six groups (5 mice per group): (1) saline (control group); (2) αPD-L1 (20 μg per mouse); (3) free GEM (5 mg/kg); (4) GEM NPs (5 mg/kg GEM equivalent); (5) GEM NPs (5 mg/kg GEM equivalent) combined with αPD-L1 (20 μg per mouse) and (6) αPD-L1/GEM NPs (5 mg/kg GEM equivalent and 20 μg αPD-L1 per mouse). The above treatments were intravenously injected every other day for a total of three times. Two days after the last injection, the mice were sacrificed and the tumor tissues were collected and homogenized. The concentration of IFN-γ in tumor tissues was determined by Mouse IFN-γ ValukineTM ELISA Kit (Novus, Bio-Techne China, catalog no. VAL607).

Flow cytometry analysis for TAA-specific T cell

After stimulated with SIINFKEL peptides as described in the experimental section (TAA-specific T-cell analysis), splenocytes were washed and stained with Zombie AquaTM (BioLegend, catalog no. 423102), Alexa Fluor 700 anti-mouse CD45 (eBioscience, catalog no. 56-9459-42), FITC anti-mouse CD3e (BioLegend, clone17A2, catalog no. 100203), PE/Cy7 anti-mouse CD4 (BioLegend, catalog no. 100422), Pacific Blue anti-mouse CD8a (BioLegend, clone 53-6.7, catalog no. 100725), APC anti-mouse IFN-γ (BioLegend, clone XMG1.2, catalog no. 505810) and PE anti-mouse CD107a (eBioscience, catalog no. 12-1071-82). The antibodies were diluted following the manufacturer’s protocol.

Table S1. Pharmacokinetics parameters of GEM and diABZI in GPS *versus* free drug combination (15 mg/kg GEM-equivalent dose, 3 mg/kg diABZI, and 200 μg αPD-L1 per rat) *in vivo*.

PK parameter	**Free GEM**	**GEM in GPS**	**Free diABZI**	**diABZI in GPS**
$t_{1/2}$ (h)	2.0±1.6	9.3±3.2	0.19±0.06	0.32±0.04
C_{max} (μg/ml)	116.0±1.8	108.4±7.4	26.0±2.0	29.9±1.8
$AUC_{(0\text{-}inf)}$ (μg·h/ml)	411.4±93.2	1381.0±160.4	6.2±0.5	18.6±1.0

* $t_{1/2}$, the half-life time of elimination phase; C_{max}, maximum drug concentration in the plasma. AUC, area under the plasma concentration-time curve. The data are presented as the means±SD (n = 4).

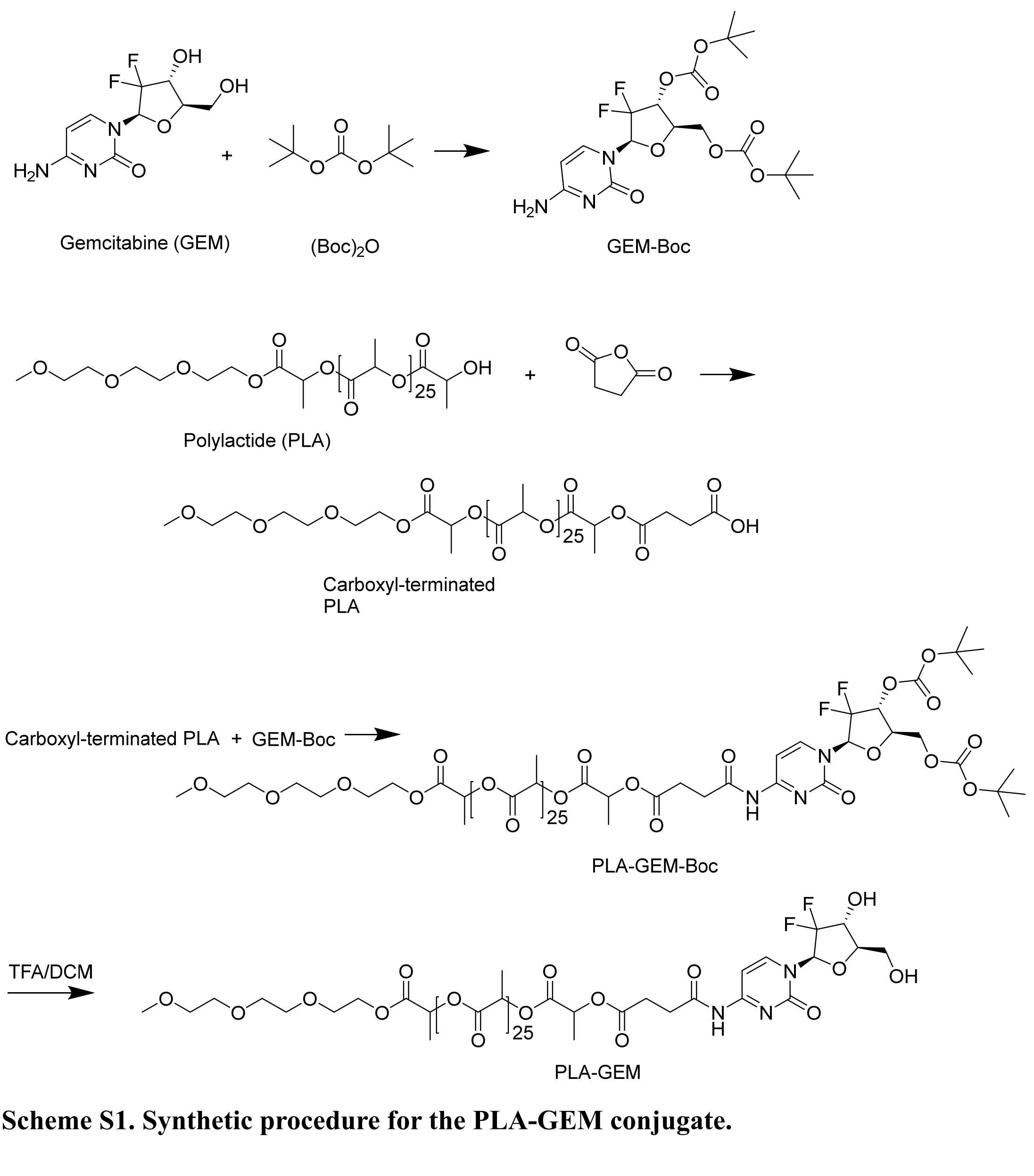

Scheme S1. Synthetic procedure for the PLA-GEM conjugate.

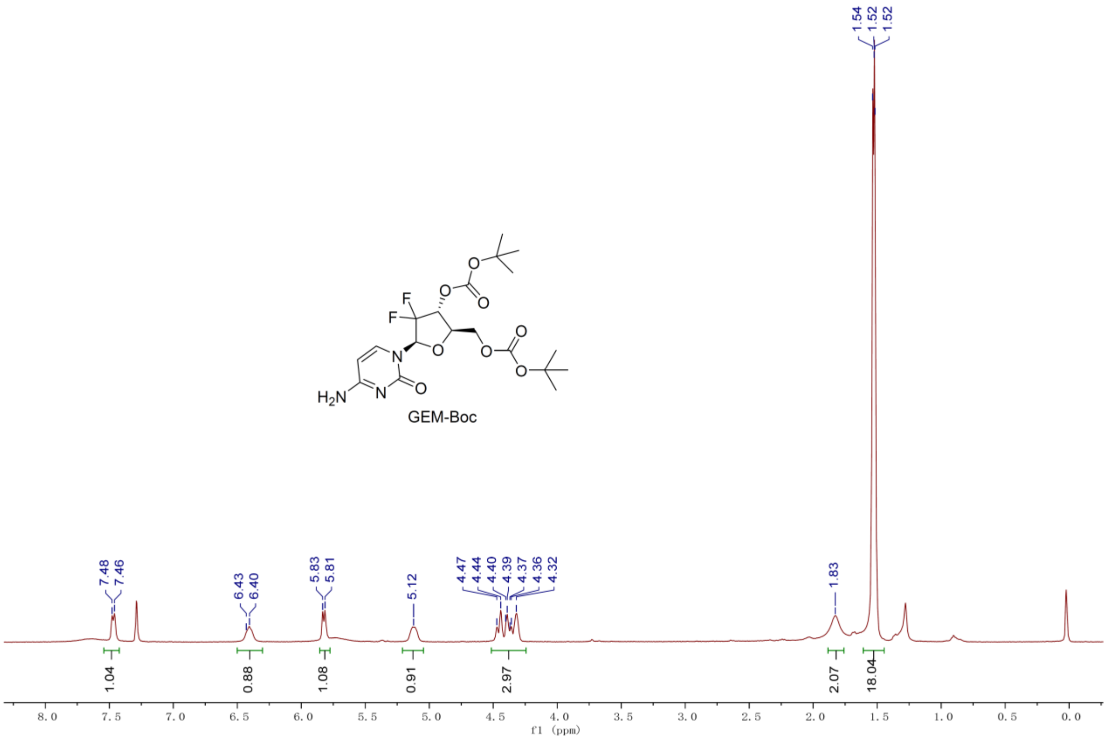
GEM-Boc
F
F
N
O
H2N
N
O
O
O
O
O
O
O
1.54
1.52
1.52
7.48
7.46
6.43
6.40
5.83
5.81
5.12
4.47
4.44
4.40
4.39
4.37
4.36
4.32
1.83
1.04
0.88
1.08
0.91
2.97
2.07
18.04
f1 (ppm)

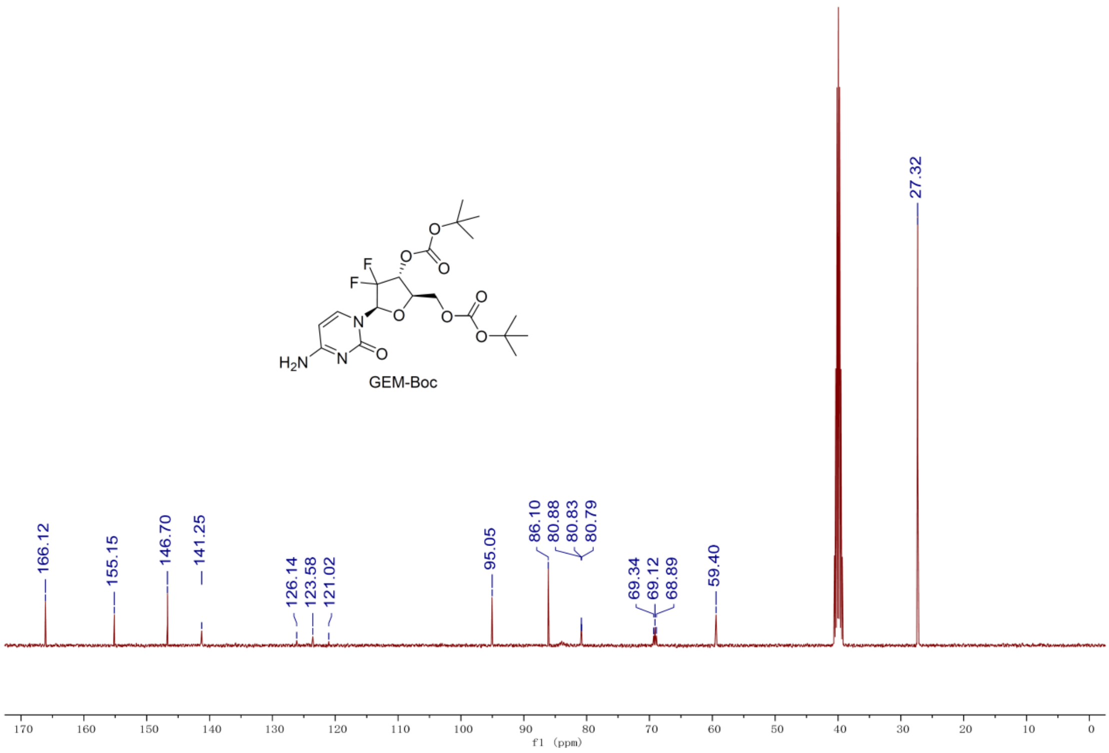
GEM-Boc
F
F
N
O
H2N
N
O
O
O
O
O
O
O
27.32
166.12
155.15
146.70
141.25
126.14
123.58
121.02
95.05
86.10
80.88
80.83
80.79
69.34
69.12
68.89
59.40
f1 (ppm)

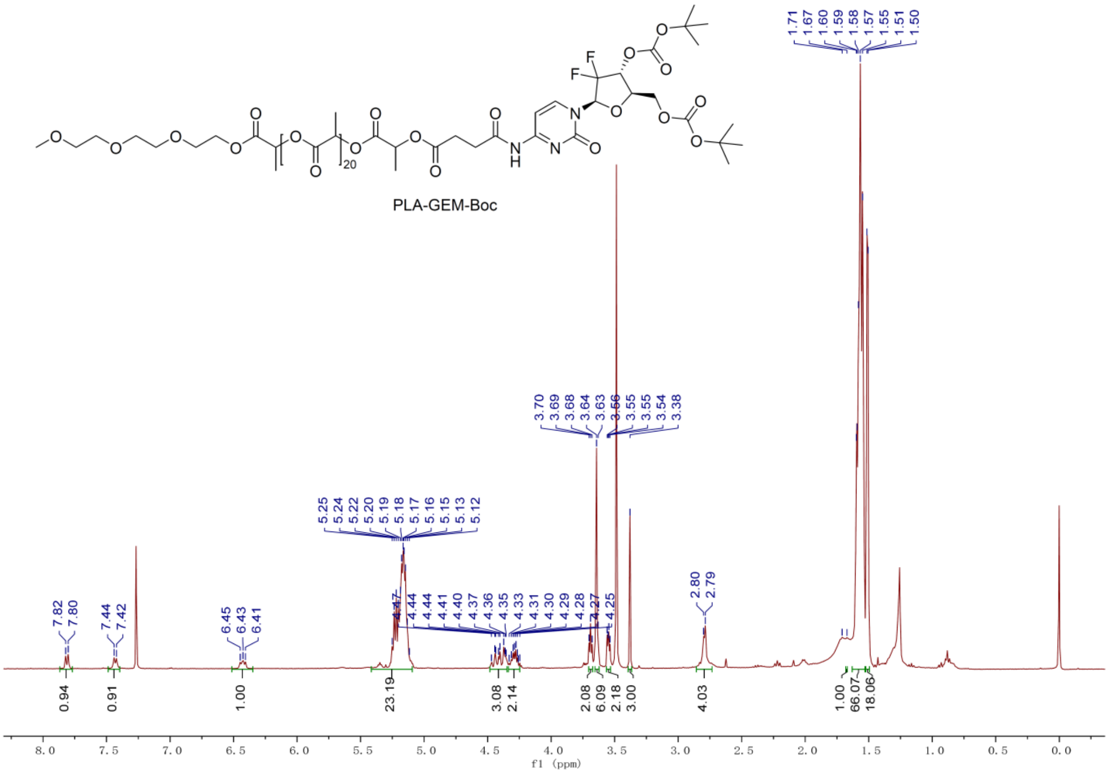
PLA-GEM-Boc
20
7.82
7.80
7.44
7.42
6.45
6.43
6.41
5.25
5.24
5.22
5.20
5.19
5.18
5.17
5.16
5.15
5.13
5.12
4.47
4.44
4.44
4.41
4.40
4.37
4.36
4.35
4.33
4.31
4.30
4.29
4.28
4.27
4.25
3.70
3.69
3.68
3.64
3.63
3.56
3.55
3.55
3.54
3.38
2.80
2.79
1.71
1.67
1.60
1.59
1.58
1.57
1.55
1.51
1.50
0.94
0.91
1.00
23.19
3.08
2.14
2.08
6.09
2.18
3.00
4.03
1.00
66.07
18.06
f1 (ppm)

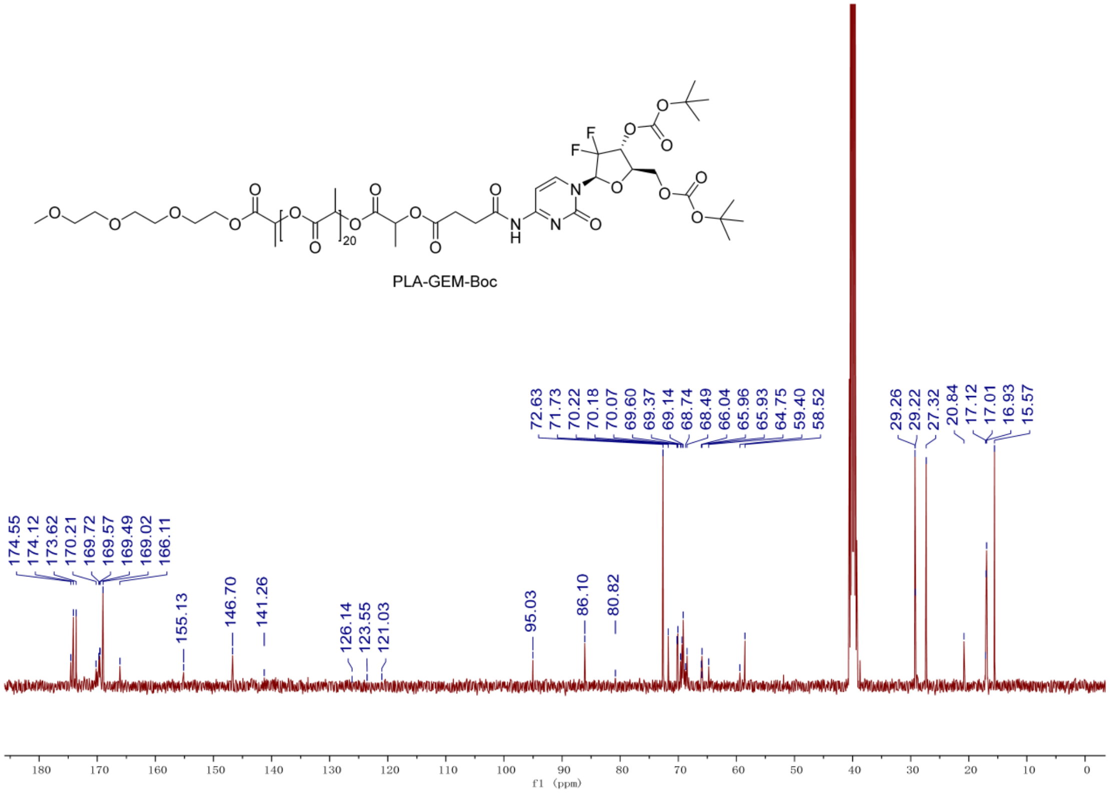
PLA-GEM-Boc
20
174.55
174.12
173.62
170.21
169.72
169.57
169.49
169.02
166.11
155.13
146.70
141.26
126.14
123.55
121.03
95.03
86.10
80.82
72.63
71.73
70.22
70.18
70.07
69.60
69.37
69.14
68.74
68.49
66.04
65.96
65.93
64.75
59.40
58.52
29.26
29.22
27.32
20.84
17.12
17.01
16.93
15.57
f1 (ppm)

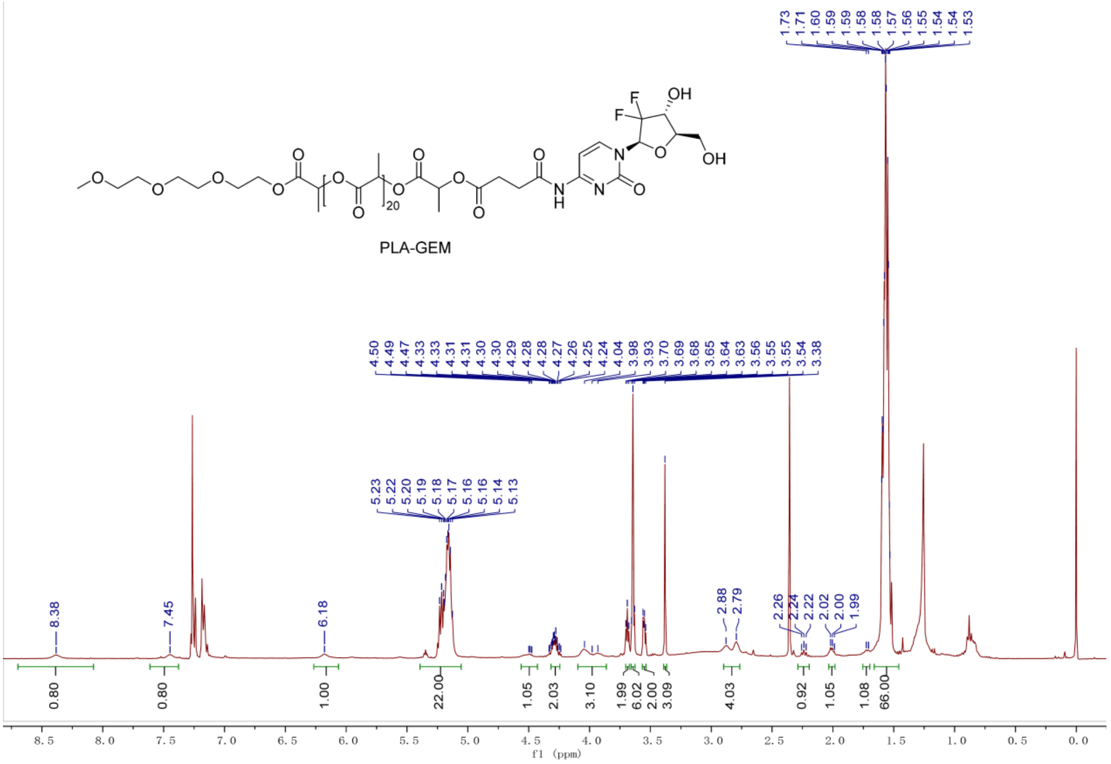

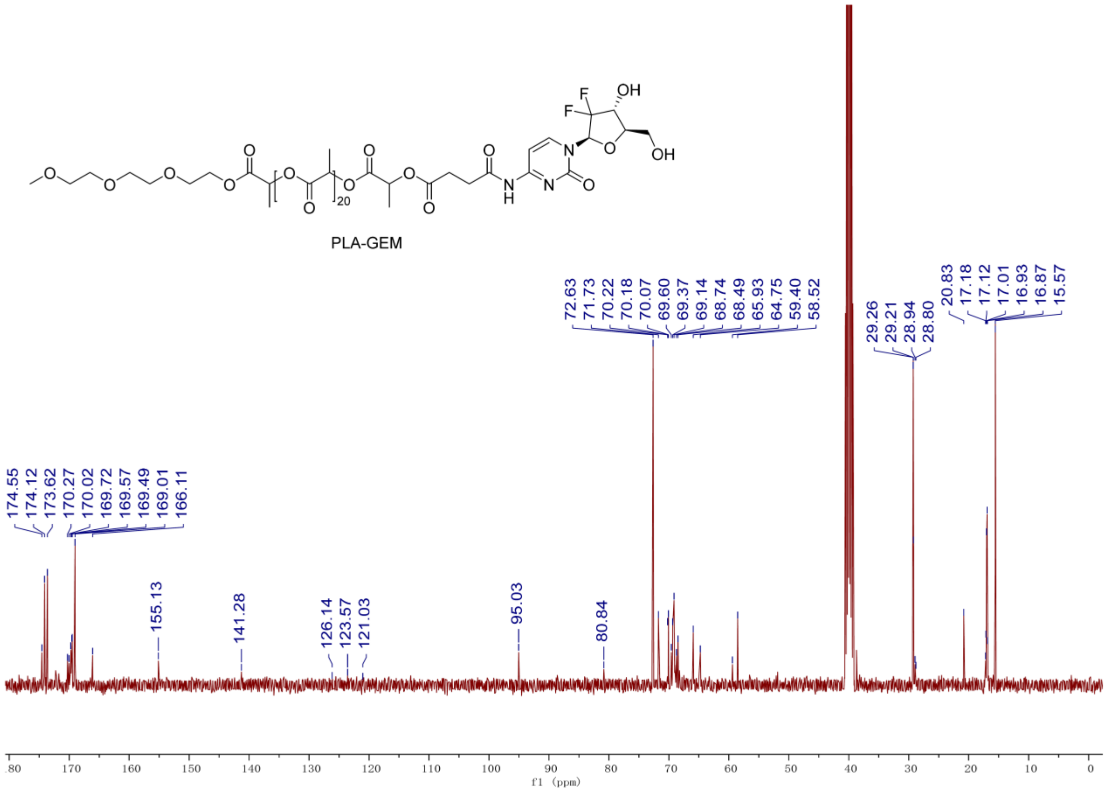

Figure S1. Characterization of GEM-Boc, PLA-GEM-Boc and PLA-GEM by the ^{1}H and ^{13}C NMR spectroscopy.

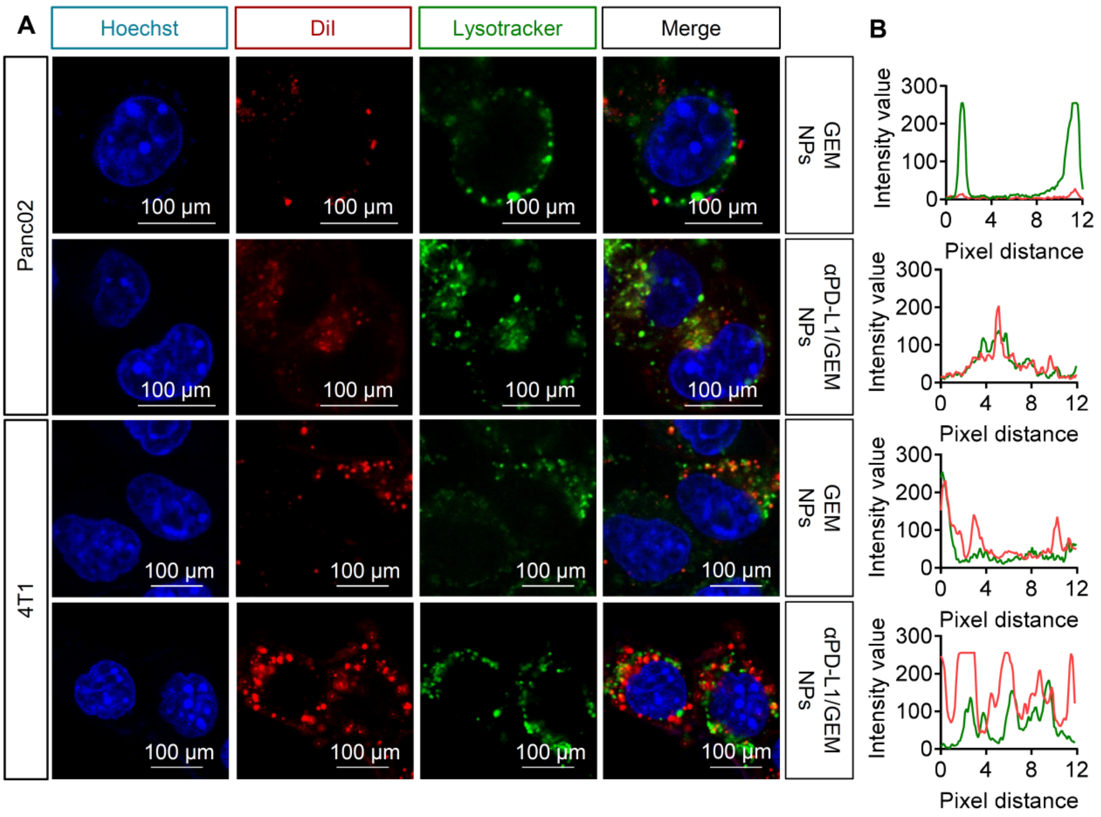

Figure S2. Cellular uptake of GEM NPs and αPD-L1/GEM NPs were visualized by confocal laser scanning microscopy (CLSM) in Panc02 and 4T1 cells. (A) Panc02 and 4T1 cells were treated with DiI-labeled GEM NPs or DiI-labeled αPD-L1/GEM NPs (at a final concentration of 30 nM GEM equivalent and 10 μM DiI dye) for 2 hours and then subjected to visualization under CLSM. Prior to observation, cell nuclei and endo/lysosomes were stained with Hoechst 33342 (blue) and LysoTracker Green NDN-26 (green), respectively. The red fluorescent signal is derived from the DiI dye. (B) Intracellular colocalization was confirmed by the concurrent fluorescence signal distribution of DiI dye and LysoTracker agent.

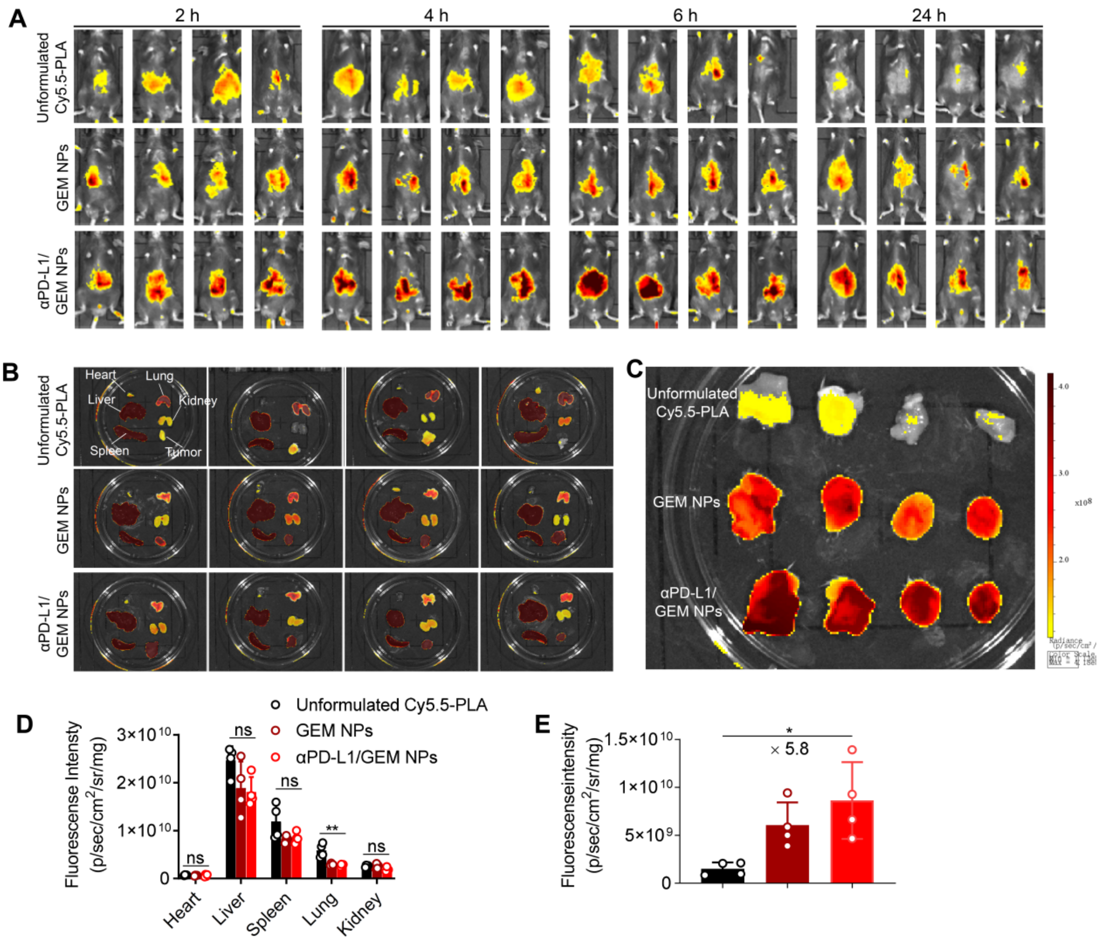

Figure S3. αPD-L1-decorated NPs achieved an enhanced accumulation in vivo. (A) C57BL/6 mice with orthotopic pancreatic cancer were intravenously injected with PLA-Cy5.5-labeled GEM NPs or αPD-L1/GEM NPs (0.75 mg/kg Cy5.5-equivalent dose). Unformulated PLA-Cy5.5 was also injected as control. The whole-body fluorescence imaging was performed under IVIS Lumina imaging system at different timepoints (2, 4, 6, and 24 h after intravenous injection). (B) Major organs (e.g., heart, liver, spleen, lung and kidney) and (C) tumors were excised at 24 h post-administration, and subjected to ex vivo imaging for the quantification of Cy5.5 fluorescence intensities. Quantitative data of the Cy5.5 fluorescence intensity from (D) excised organs and (E) tumors obtained from (B) and (C), respectively. Data shown are mean ± s.e.m. (n=4). Statistical analysis was performed with one-way ANOVA test. *p<0.05, **p<0.01, and ***p<0.001.

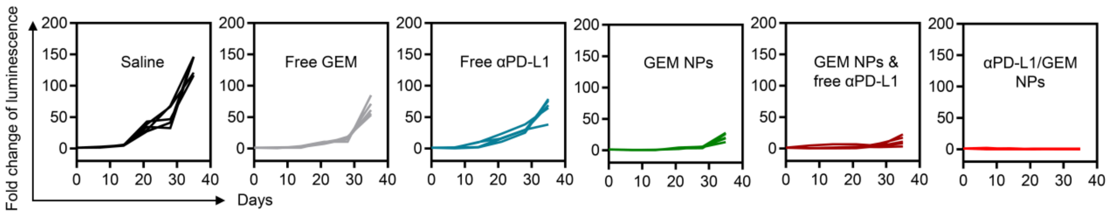

Figure S4. Antitumor efficacy of αPD-L1/GEM NPs in an orthotopic pancreatic cancer model. Individual tumor growth curves for the different treatment groups (n = 5). Tumor growth was quantified by the total luminescence flux as measured with an IVIS imaging system. The intensity fold change (Y axis) was normalized to the average luminescence flux on day 0.

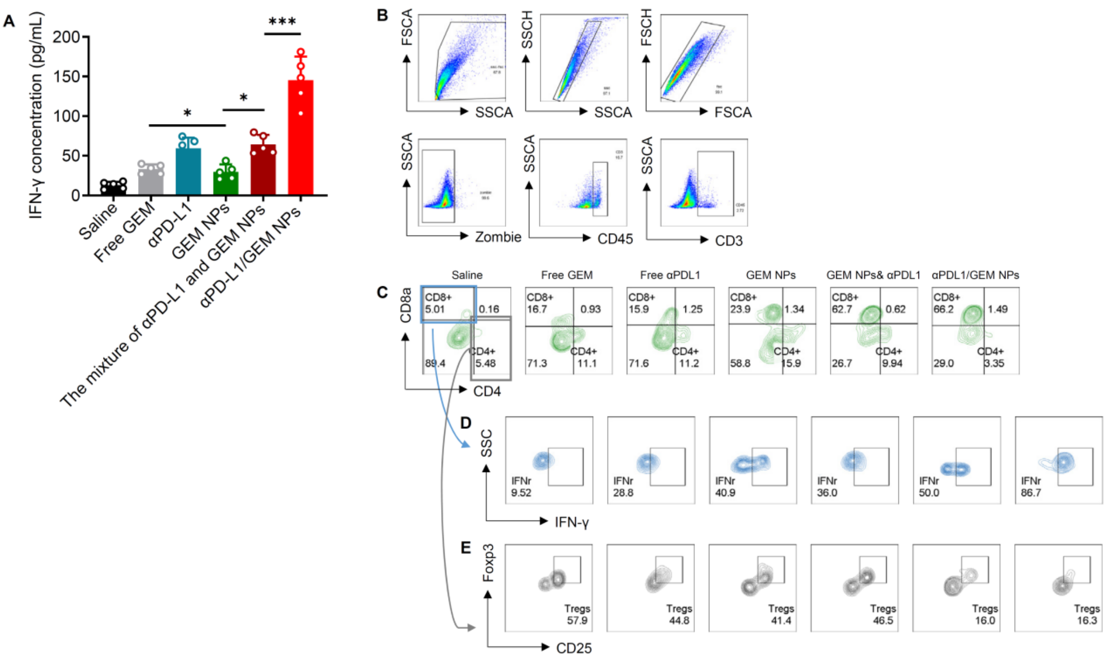

Figure S5. Flow cytometry analysis and ELISA test for the orthotopic pancreatic tumor model. (A) The concentration of IFN-γ within tumor tissues across different treatment groups was determined by ELISA test. (B) Gating strategy for T cell populations. (C) Flow cytometry plots for $CD8^+$/$CD4^+$ T cells. The upper left area indicates the proportions of $CD8^+$ T cells and lower right area indicates the proportions of $CD4^+$ T cells. (D) Flow cytometry plots for IFN-γ^+ $CD8a^+$ T cells across different treatment groups. (E) Flow cytometry plots for $CD25^+$ $Fxop3^+$ Treg cells across different treatment groups. Statistical analysis was performed with one-way ANOVA test. *p<0.05, **p<0.01, and ***p<0.001.

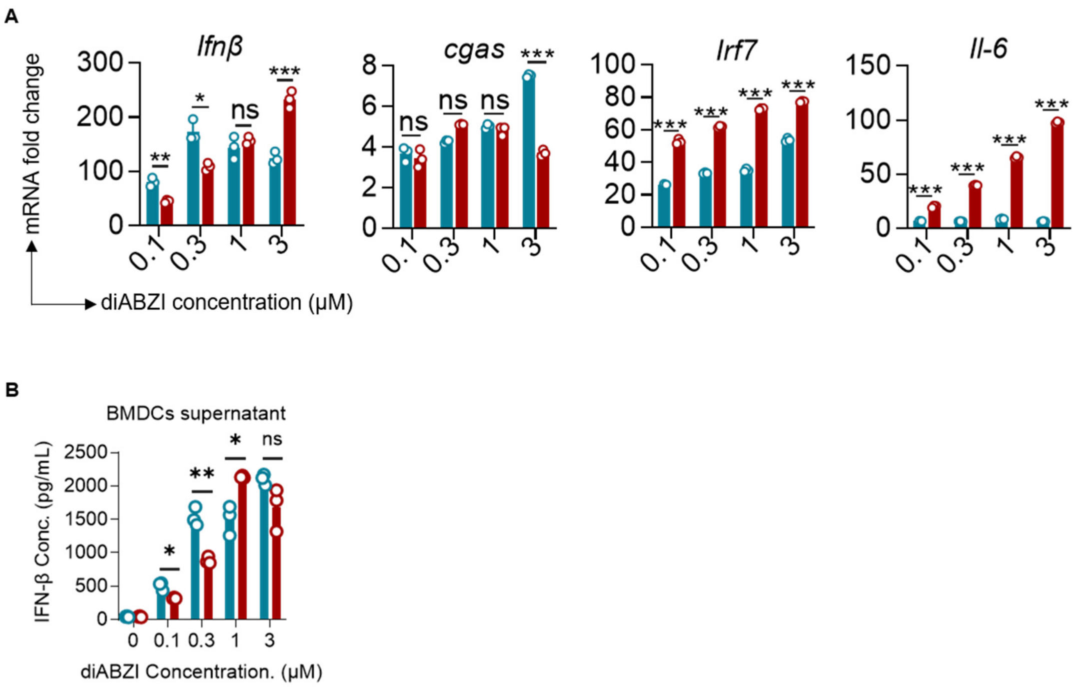

Figure S6. Nanodelivery of diABZIs preserved its high immunostimulatory activity in BMDCs. (A) PCR analysis of IFN-β, cGAS, IRF7 and IL-6 gene expression in BMDMs after treatment with diABZI or physical mixture of free diABZI, GEM and αPD-L1 (referred as 3 Free) for 4 hours. The X axis indicates the equivalent dose of diABZI. (B) IFN-β concentration in supernatants of BMDCs exposed to the above mentioned treatments for 24 hours. Statistical analysis was performed with one-way ANOVA test. *p<0.05, **p<0.01, and ***p<0.001.

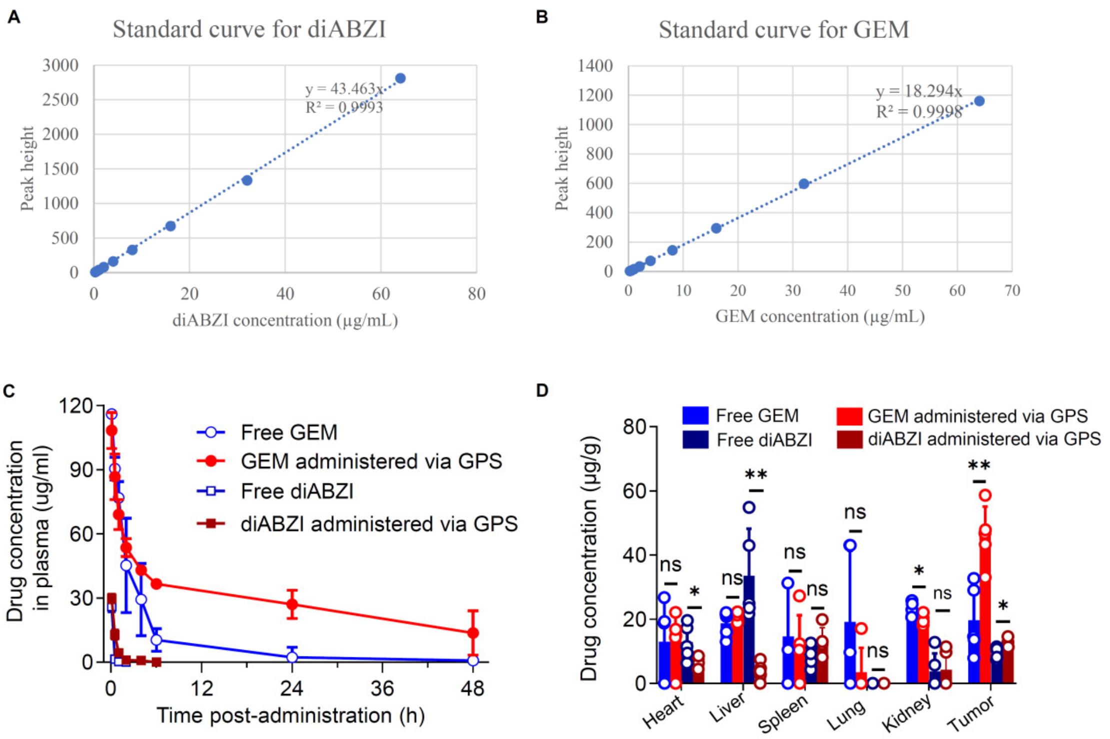

Figure S7. Pharmacokinetic and biodistribution analyses of GPS and free combination. (A-B) Standard curves for (A) diABZI and (B) GEM quantified by HPLC analysis. Y axis indicates the peak height and X axis indicates the concentration of GEM or diABZI (μg/mL). (C) Pharmacokinetic studies were performed in Sprague–Dawley (SD) rats. After a single injection of GPS or 3 free (15 mg/kg GEM-equivalent dose, 3 mg/kg diABZI and 200 μg αPD-L1), the plasma concentrations of GEM and diABZI at different timepoints were determined by HPLC analysis. (D) Biodistribution studies were performed in C57BL/6 mice bearing Panc02 orthotopic pancreatic tumors. After a single injection of GPS or 3 free (15 mg/kg GEM-equivalent dose, 3 mg/kg diABZI and 20 μg αPD-L1 per mouse), major organs and tumors were collected and drug concentration was quantified by HPLC analysis.

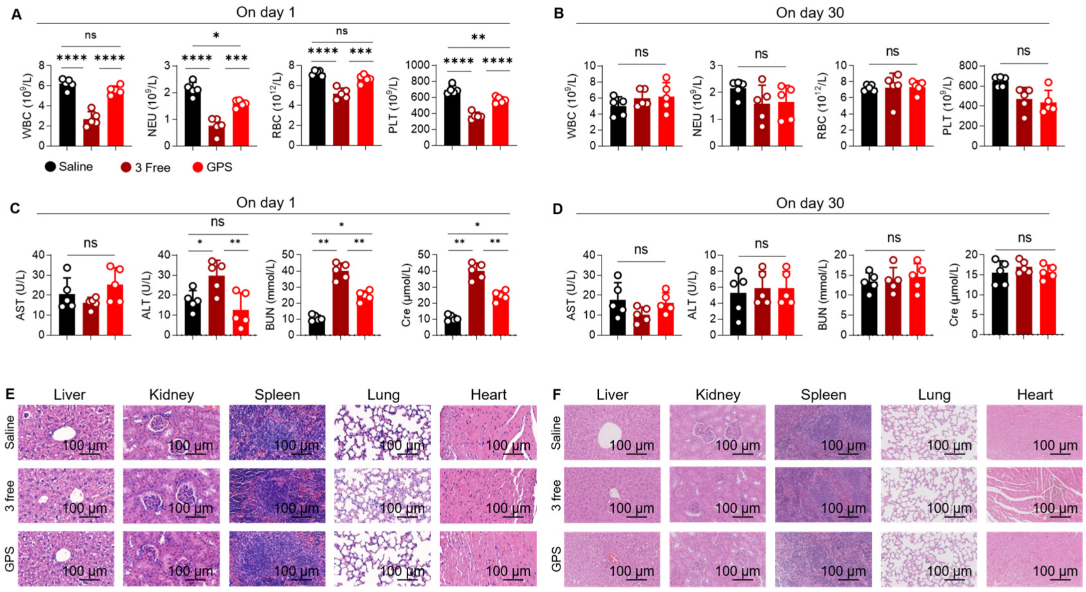

Figure S8. Safety profiles of GPS and free drug combination therapies in healthy ICR mice. (A-B) After injection of GPS or 3 free (15 mg/kg GEM-equivalent dose, 3 mg/kg diABZI and 20 µg αPD-L1 per mouse), blood was collected on day 1 (A) and day 30 (B) post-injection for blood routine test. The changes of white blood cells (WBC), neutrophils (NEU), red blood cells (RBC) and platelet (PLT) numbers were presented as statistical charts. (C-D) After injection of GPS or 3 free (15 mg/kg GEM-equivalent dose, 3 mg/kg diABZI and 20 µg αPD-L1 per mouse), blood was collected on day 1 (C) and day 30 (D) post-injection for blood biochemistry test. The changes of serum aspartate aminotransferase (AST), alanine aminotransferase (ALT), blood urea nitrogen (BUN), and creatine (Cre) were presented as statistical charts. (E-F) Histopathological examination of major organs on day 1 (E) and day 30 (F) post-injection. Scale bar: 100 µm.

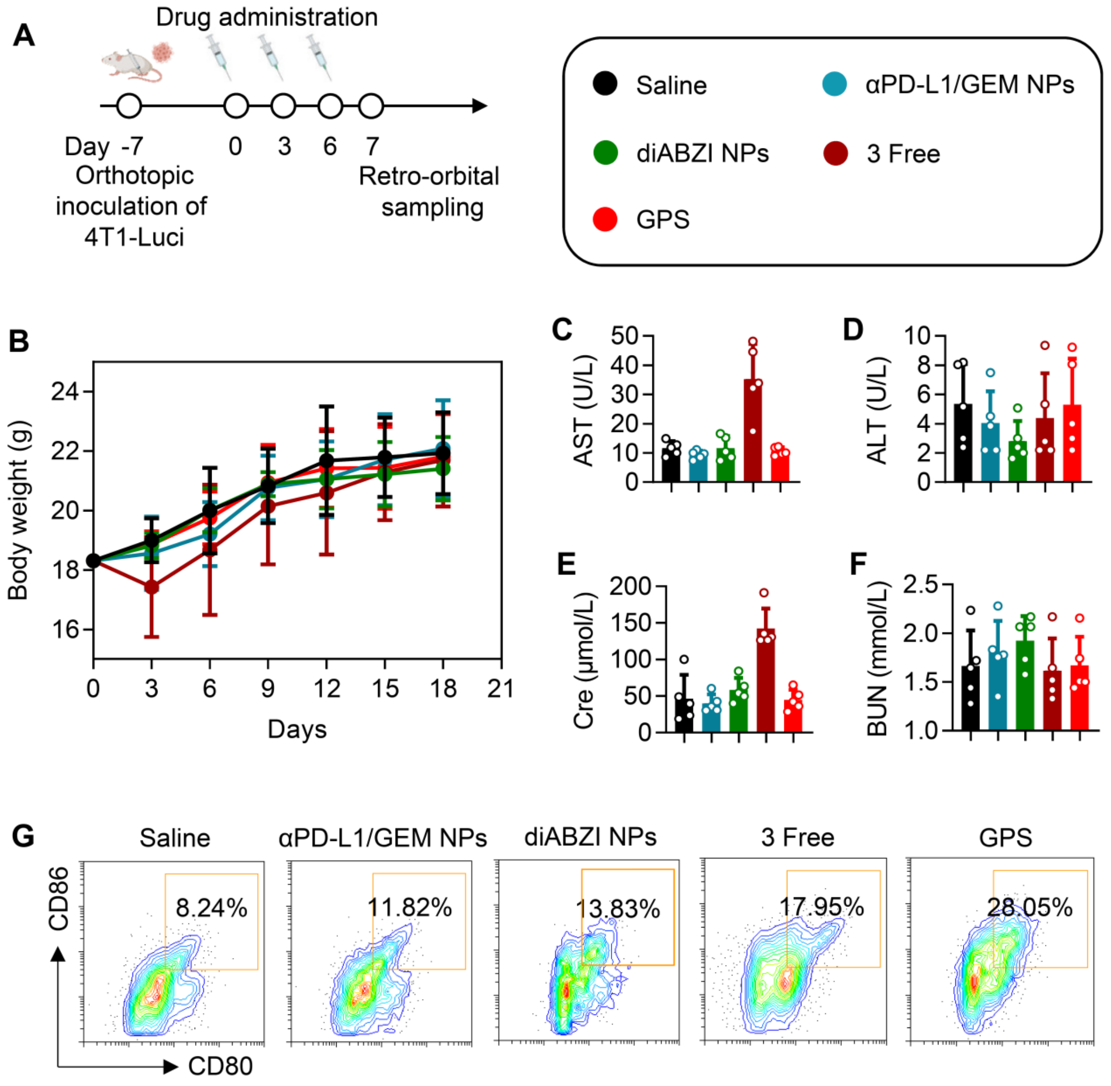

Figure S9. Safety profiles of GPS NPs in 4T1 breast cancer model. (A) A schematic of the experimental timeline and different treatment groups. (B) Body weight was recorded at different time points. (C-F) blood was collected 24 h after the last drug injections. Biochemical indicators including serum (C) AST, (D) ALT, (E) creatine and (F) BUN were evaluated for safety profile. (G) Flow cytometry plots for CD80^{+}CD86^{+} DCs (distributed in upper right quadrant in flow cytometry plots) isolated from TILs. The tumors were collected from the 4T1 breast cancer model on day 2 after a total of three injections with different treatments. Data shown are mean ± s.e.m. (n = 5).

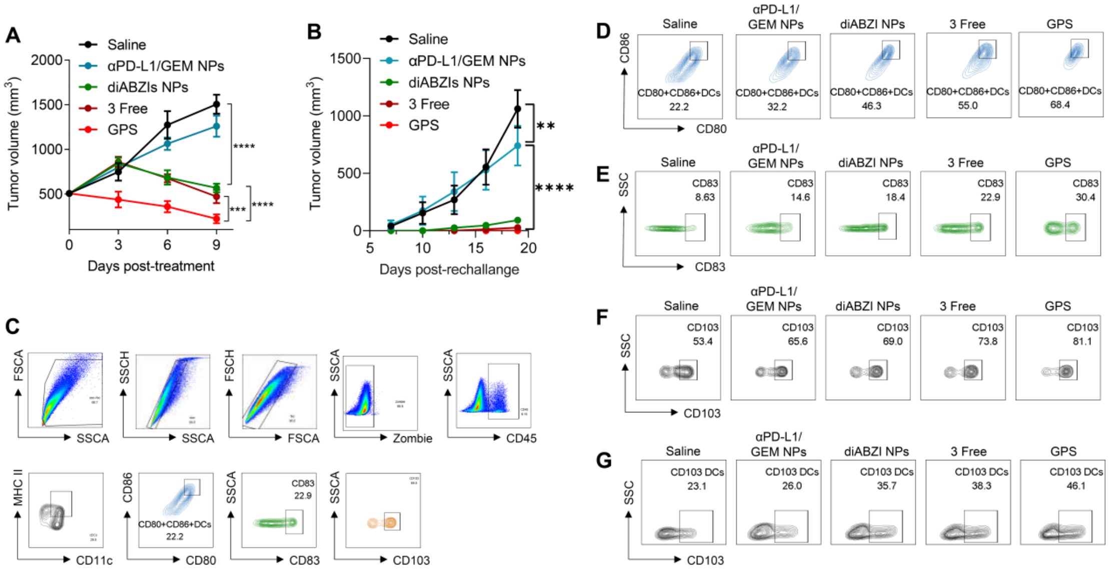

Figure S10. Melanoma growth curve and flow cytometry plots for DC maturation. (A-B) The relative volumes of (A) primary and (B) rechallenged melanoma tumors were determined by different time points. (C) Gating strategy for DC populations. (D-E) Flow cytometry plots for (D) $CD80^{+}/CD86^{+}$ and (E) $CD83^{+}$ DCs isolated from TILs. The tumors were collected from the B16-OVA melanoma model on day 2 after a total of three injections with different treatments. (F-G) Flow cytometry plots for $CD103^{+}$ DCs isolated from (F) TILs and (G) tdLNs. The tumors and tdLNs were collected from the B16-OVA melanoma model on day 2 after a total of three injections with different treatments.

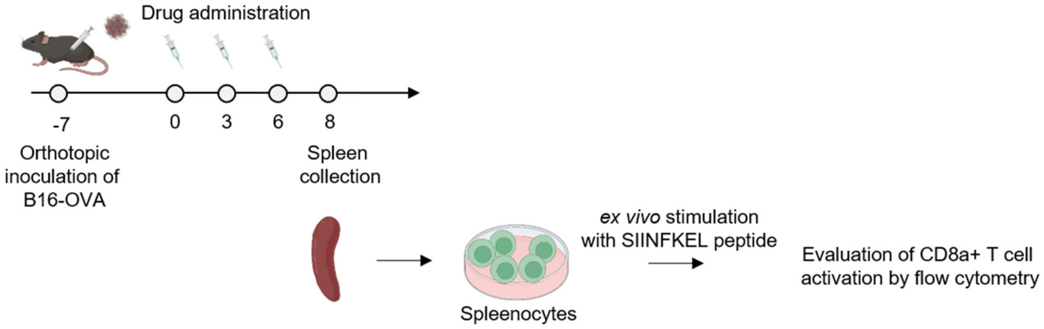

Figure S11. Schematic illustration of TAA-specific T-cell analysis. Spleens from B16-OVA tumor-bearing C57BL6/J mice were collected on day 2 after the last injection of the corresponding treatment. Splenocytes were stimulated with the SIINFKEL peptide for 6 hours. $CD8a^{+}$ T cell activation was analyzed by flow cytometry.
